# Phenolic Composition in Native and Defatted Nuts and Seeds from the Serbian Market: Analytical Insights and Functional Potential

**DOI:** 10.3390/foods14244191

**Published:** 2025-12-06

**Authors:** Margarita Dodevska, Nevena Ivanović, Sanja Đurović, Boris Pisinov, Uroš Čakar, Jelena Kukić Marković

**Affiliations:** 1 Institute of Public Health of Serbia “Dr Milan Jovanovic Batut”, Dr Subotića 5, 112113 Belgrade, Serbia; margarita_dodevska@batut.org.rs; 2 Department of Bromatology, University of Belgrade-Faculty of Pharmacy, Vojvode Stepe 450, 11221 Belgrade, Serbia; nevena.ivanovic@pharmacy.bg.ac.rs (N.I.); uros.cakar@pharmacy.bg.ac.rs (U.Č.); 3 Institute for Plant Protection and Environment, Teodora Drajzera 9, 11040 Belgrade, Serbia; stojakovicsm@yahoo.com (S.Đ.); boriss752002@yahoo.com (B.P.); 4 Department of Pharmacognosy, University of Belgrade-Faculty of Pharmacy, Vojvode Stepe 450, 11221 Belgrade, Serbia

**Keywords:** hydroxycinnamic acids, hydroxybenzoic acids, phenolics, flavonoids, HPLC-DAD, PCA

## Abstract

In this study the phenolic profile of 25 different commercially available edible nuts and seeds from the Serbian market and their defatted by-products were analyzed and compared. Results showed that both native and defatted nuts and seeds are rich sources of various phenolics. Of all the samples analyzed, walnuts, pecan nuts and sunflower seeds (raw and roasted) showed the highest total phenolic content. Sunflower seeds, especially the raw ones, proved to be an exceptionally rich source of chlorogenic acid (116,928.66 μg/g), exceeding the values previously reported in the literature. Similarly, walnut and pecan samples showed the highest levels of protocatechuic and gallic acids, while high flavonoid concentrations in different peanut samples and chia seeds suggest that these commonly consumed foods may have greater bioactive potential than previously thought. The present research confirmed the fact that certain edible nuts and seeds as well as their defatted by-products, already valued for their nutritive values, are affordable, sustainable and rich natural sources of various bioactive phenolics, especially phenolic acids. This work substantiates data on phenolic profiles of edible nuts and seeds, strengthening the foundation for the development of functional foods and contributing to the valorization of agro-industrial residues in line with the principles of circular economy and functional food innovation. Our results also highlight some important and relatively constant characteristics of phenolic composition and content in certain nuts and seeds. These characteristics could potentially serve as quality parameters for the respective samples, enabling the development of products with uniform and standardized composition, one of the prerequisites for high-quality products with pronounced activity.

## 1. Introduction

A wide range of phytochemicals, including various phenolics, are present in many edible nuts and seeds such as almonds, walnuts, hazelnuts, pecans, cashews, linseeds and sesame seeds. The presence of these compounds, along with essential fatty acids, proteins, fibers, carbohydrates, vitamins and minerals, makes them important functional food constituents [1,2,3]. Significant amounts of defatted by-products are obtained through mechanical or solvent-based extraction of edible oils from different nuts and seeds. These so-called oilcakes or meals are rich in protein, fiber, essential micronutrients and bioactive compounds with health-promoting properties [4]. With growing environmental and nutritional awareness, the utilization of these agro-industrial residues has gained momentum as a sustainable strategy in line with circular economy goals [5,6]. In addition to their nutritional and functional potential, the incorporation of oilseed cakes into food systems contributes to food security, reduces waste and supports the development of value-added products—thereby reinforcing sustainable agricultural practices and contributing to broader health and sustainability goals [6].

Studies on the chemical composition of certain oilseed cakes are substantial and do not only consider components with nutritional value, but focus more on bioactive compounds such as various phenolics, triterpenes and other secondary plant metabolites that exhibit a range of health-promoting properties [7]. For example, sunflower seed, sesame and pecan nuts cakes showed high amounts of total extractable phenolics and high antioxidant activity, implicating their beneficial effects on health, especially against chronic non-communicable diseases [8,9,10].

Though secondary metabolites are not essential for plant growth and development, they play an important role in their relationship with the environment, e.g., in plant defense and adaptation to various factors [11]. These secondary metabolites include phenolic acids as ubiquitous phenolic compounds. They can be found in many different plant foods and are consumed daily, especially in vegetables, fruit, cereals, spices and beverages such as tea and coffee. There are two main groups of phenolic acids, hydroxybenzoic acids (HBAs) and hydroxycinnamic acids (HCAs), with HCAs usually being more prevalent than HBAs. It is estimated that phenolic acids constitute about one-third of the total dietary phenolics in plant-based foods [12].

Phenolic acids from food are easily accessible and exhibit various health-promoting activities (antioxidant, anti-inflammatory, antitumor, antimicrobial, analgesic, antidiabetic, cardio- and neuroprotective). In addition to their importance as functional food ingredients, there is a growing interest in the use of phenolic compounds, including phenolic acids, in many other areas, e.g., in the food industry, cosmetology and biotechnology, as antioxidants, preservatives, bioactive packaging ingredients, etc. [13].

Investigations into the composition and content of phenolic compounds in various nuts, seeds, and their by-products are increasing, yet considerable variability among published datasets persists. According to the literature, such fluctuations are often attributed to differences in cultivar, ripeness, growing conditions, and pre- or post-harvest handling of plant material [14,15]. However, this information is generally not available for commercially sourced products. Another reason is the use of different extraction techniques and analytical methods for investigation of chemical composition and biological potential. Also, a huge number of investigations refer to the optimization of the extraction process in order to obtain products with the highest usability in terms of maximizing the content of phenolics or some other ingredients and thus pronounced bioactivity [7,16,17,18,19,20,21].

Previous studies investigating phenolic compounds in nuts, seeds, and their by-products typically analyze either native kernels or defatted press cakes, while studies characterizing both fractions simultaneously are comparatively rare. When such studies are available, they generally include native and defatted samples of only one nut or seed type, rather than a broader range of commercially relevant species [22]. As a result, comprehensive profiling of commercially available nut- and seed-based products and their by-products remains limited, and to date, no systematic analysis has been conducted for products available on the Serbian market. Addressing this gap is essential for generating realistic baseline composition data that reflect foods as consumed in Serbia, support dietary exposure assessments, and inform the development of functional foods and circular-economy-oriented valorization strategies [1,17,18,21].

Therefore, the aim of this study was to comprehensively characterize the phenolic composition of native and defatted fractions of nuts and seeds available on the Serbian market. By analyzing both matrices in parallel, the study provides comparable and realistic composition data relevant to understanding the phenolic profiles of commonly consumed products and to supporting the valorization of nut- and seed-derived by-products in functional food applications.

## 2. Materials and Methods

### 2.1. Chemicals

Analytical standards (purity ≥ 98%) were purchased from Sigma-Aldrich Chemie GmbH (Merck KGaA, Darmstadt, Germany): *trans*-cinnamic acid, *p*-coumaric acid, caffeic acid, *trans*-ferulic acid, chlorogenic acid, dihydrocaffeic acid, phloretic acid, hesperetic acid, syringic acid, protocatechuic acid, catechin, epicatechin, esculin, naringin, naringenin, quercetin; gallic acid and rutin were purchased from Fluka (Buchs, Switzerland); synapic acid was purchased from Extrasynthese (Genay, France). They were used for identification and quantification of phenolic compounds. Analytical-grade methanol CHROMASOLV™ (≥99.9%) and HPLC-grade water CHROMASOLV Plus were purchased from Riedel-de Haën (Honeywell, Charlotte, NC, USA). All other solvents and chemicals were p.a. or higher purity.

### 2.2. Samples and Extraction

Twenty-five different types of nuts and seeds (native samples) were purchased from several local markets in Belgrade, Serbia (Table 1). Most samples were purchased as bulk, non-prepacked nut and seed products from retail outlets in Belgrade, Serbia, including specialized stores and open-scoop “bulk bin” shops, which are common and affordable sources of nuts and seeds for the Serbian population. For each sample, the nut or seed type, processing form (raw, roasted, boiled, or marinated), and purchase date were recorded. All samples were transferred to the laboratory in paper bags and kept at +4° C till analysis. The native samples were finely ground in an electrical mill (SM-450; MRC, Holon, Israel) and then defatted using dichloromethane. One portion of the native samples was extracted with dichloromethane (1:10, *w*/*v*) by maceration for 24 h, on a laboratory shaker (KS 15A; Edmund Büchler GmbH, Hechingen, Germany). The extracts were filtered and defatted, and residual plant material was dried (M sample). Another portion of the native sample was extracted with dichloromethane (1:10 *w*/*v*) in a Soxhlet apparatus, over 3 h, and the obtained defatted residue was also dried (S sample). Finally, one portion of the native sample and both dry defatted samples obtained were extracted each with 80% methanol (1:10, *w*/*v*) by maceration for 24 h on a laboratory shaker. The obtained hydromethanol extracts were filtered and dried (N, M and S, respectively) and further analyzed.

### 2.3. Total Phenolic and Flavonoid Contents

Total phenolic content (TPC) was determined using Folin–Ciocalteu (FC) reagent according to Ušjak et al. [23] and expressed as gallic acid (GA) content (µg GA/mg), while total flavonoid content (TFC) was measured based on the reaction between flavonoids and aluminum chloride and expressed as catechin (C) content (µg C/mg) [24]. All measurements were conducted in triplicate.

### 2.4. HPLC-DAD Analysis

The identification and quantification of phenolic compounds (HBAs, HCAs, flavonoids and coumarins) was performed on a Shimadzu Nexera XR liquid chromatograph (Shimadzu Corporation, Kyoto, Japan) equipped with an autosampler, using a previously described and validated method [25]. Separation was carried out on an Agilent Zorbax SB C18 column (250 × 4.6 mm, id. 5 μm, Agilent Technologies Inc., Santa Clara, CA, USA), thermostatically controlled at 25 °C. The flow rate was set to 1 mL/min. The two-component mobile phase consisted of A (0.1% HCOOH in water) and B (methanol) and was used in a gradient mode in volumetric ratios as follows: 0 min 5% B, 25 min 30% B, 35 min 40% B, 40 min 48% B, 50 min 70% B, 55 min 100% B, 65 min 5% B, re-equilibration time 10 min. Dual wavelengths (280 and 325 nm) were used to detect the eluted compounds. Identification of phenolic compounds was performed by comparing their UV spectra and retention time to those of standard compounds (Table S1). The external standard method was used for quantification. A stock solution of standards of known purity was prepared in a methanol–water mixture (75:25, *v*/*v*) at a concentration of 1.0 mg/mL. The working solutions were prepared by diluting the stock solution with a methanol–water mixture to concentrations of 25, 50, 75, 100, 125, and 150 µg/mL. The concentrations of individual phenolic compounds were calculated using calibration curves of the corresponding standards. Dry samples (N, M, S) were redissolved in 80% methanol, filtered through 0.45 µm PTFE syringe filters and subjected to HPLC analysis. HPLC analyses were performed in duplicate, and the results are expressed as µg per g of dry matter (µg/g d.m.). LabSolutions software (Shimadzu Corporation, Kyoto, Japan) was used to calculate the concentration of individual phenolic compounds in the samples. The method quality parameters (LOD (limit of detection), LOQ (limit of quantification), linearity, and correlation coefficient) are given in Table S1. The linear range of the tested phenolic compounds was 10–500 µg/mL.

### 2.5. Statistical Analysis and Principal Component Analysis

The statistical data analysis was performed using SPSS 20.0 (IBM Corp, Armonk, NY, USA) and Microsoft Excel 2013 (Microsoft, Redmond, WA, USA). One-way analysis of variance (ANOVA) was employed to compare different types of extraction within groups. For the individual comparisons, the least significant difference (LSD test) was used. Principal component analysis (PCA) was employed to observe correlations between analyzed parameters (elements) and examine similarities between the individual nut and seed samples. Biplots were constructed to achieve visualization. The correlation analyses between phenolic derivatives and the total amount of secondary metabolites were conducted using the Pearson correlation test.

## 3. Results

Hydromethanol extracts of different commercial samples of nuts and seeds (a total of 75 samples, native and defatted ones) were analyzed for their total phenolic content (TPC) and total flavonoid content (TFC). At the same time, their phenolic profile (HBAs, HCAs, flavonoids and coumarins) was determined using the HPLC-DAD technique.

The TPC in hydromethanol extracts of native samples showed that walnut, pecan nuts and sunflower seeds (raw and roasted) had the highest TPC values (150.1, 147.9, 98.4 and 93.3 µg GA/mg, respectively), while in almond samples, the TPC was the lowest (3.1–6.8 µg GA/mg) as well as in pine nuts (5.4 µg GA/mg; Table 1). Among defatted samples, the highest TPC was also measured in walnut (155.3 and 132.5 µg GA/mg in M and S samples) and pecan samples (140.1 µg GA/mg in M sample). All defatted samples of sunflower seeds had significantly higher TPC values compared to N samples.

As for TFC, the results were somewhat different. The highest values were determined in N samples of roasted and raw sunflower seeds (121.3 and 103.3 µg C/mg, respectively), followed by pecan nuts (61.3 µg C/mg). Significant TFC was measured in walnut, raw peanuts and chia seeds (27.6, 23.6 and 34.0 µg C/mg). Native samples of hemp and pumpkin seeds had the lowest TFC (below 1 µg C/mg). Among defatted samples, the highest TFC was determined in M and S samples of raw sunflower seeds (157.4 and 147.1 µg C/mg, respectively) and M and S samples of roasted sunflower seeds (101.7 and 120.5 µg C/mg, respectively). The TFC of other defatted samples was substantially lower (Table 1).

**Figure 1:**
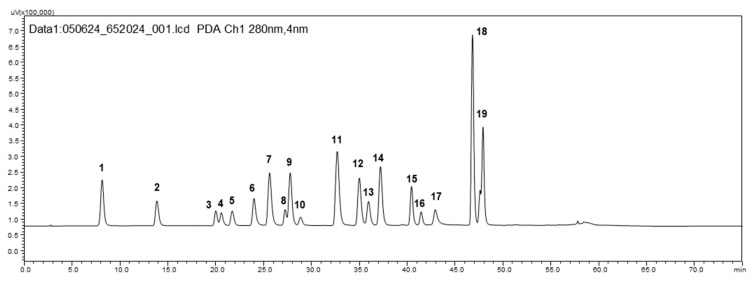
HPLC-DAD chromatogram of standard compounds (100 µg/mL) at 280 nm (1—gallic acid; 2—protocatechuic acid; 3—esculin; 4—catechin; 5—dihydrocaffeic acid; 6—chlorogenic acid; 7—caffeic acid; 8—epicatechin; 9—syringic acid; 10—phloretic acid; 11—*p*-coumaric acid; 12—ferulic acid; 13—synapic acid; 14—hesperetic acid; 15—naringin; 16—rutin; 17—*trans*-cinnamic acid; 18—quercetin; 19—naringenin).

HPLC-DAD analysis of phenolic (Figure 1) content in the investigated native samples showed that walnut samples exhibited high phenolic content, especially HBAs (9717.2 µg/g). The same trend was observed for defatted walnut samples, with the highest HBA content (15,809.5 and 11,445.4 µg/g in M and S, respectively) among all other tested samples. Raw and roasted sunflower seeds apparently were the richest sources of HCAs (120,429.6–137,230.6 and 89,629.7–107,533.1 µg/g, respectively). Significant HCA content was also found in walnut and sesame samples (4591.2–5466.5 and 4626.1–6075.2 µg/g, respectively). The highest flavonoid content was observed in linseed samples (71,882.6–83,382.3 µg/g), while in other investigated samples, it was a few times lower. Still, sesame, walnut, chia and all peanut samples also contained substantial amounts of flavonoids (ranging from ca. 9000 to over 15,000 µg/g). As for coumarins, the highest content was determined in chia samples (2065.4–3238.7 µg/g), followed by hemp seed samples (741.9–979.5 µg/g; Table 2).

On the other hand, native linseed samples along with native pine nut samples were characterized by the lowest HBA content (286.8 and 390.9 µg/g, respectively). The same could be stated for their respective defatted samples. The content of HCAs in pine nut samples (229.3–273.4 µg/g) was also the lowest among all samples investigated. As for flavonoids, their content was the lowest in hemp seed samples (963.8–1011.3 µg/g) (Table 2).

In terms of individual phenolic contents (Table 3), walnut samples contained the highest amount of protocatechuic (5775.9–11,551.8 µg/g) and gallic (2844.0–5203.2 µg/g) acids and a substantial amount of syringic acid (398.2–503.64 µg/g). Samples with high gallic acid content were pecan (1290.0–1315.5 µg/g) and roasted and raw pumpkin seeds (1149.4–1705.4 and 910.7–1627.5 µg/g). Syringic acid was the least represented HBA in the investigated samples. The main source of this compound seems to be raw and roasted sunflower seeds (1269.6 and 624.7 µg/g in N samples, respectively). Besides the aforementioned walnut samples, all peanut samples contained substantial quantities of syringic acid compared to other samples (Table 3).

As for HCAs (Table 4), sunflower seeds appear to be the richest sources of chlorogenic acid, containing 87,715.1 µg/g (roasted sunflower seed N sample) to 13,3467.1 µg/g (raw sunflower seed S defatted sample). Raw and roasted peanuts were significant sources of *p*-coumaric acid (288.3–940.0 and 845.5–1093.3 µg/g, respectively) while linseed samples contained a substantial amount of cinnamic acid (2446.3–2998.0 µg/g, respectively). Ferulic acid was also dominant in raw sunflower seed samples (1392.2–1607.8 µg/g). Dihydrocaffeic acid was the main HCA in walnut and pecan samples (2477.6–3294.3 and 2558.6–3068.6 µg/g, respectively).

In the flavonoid fraction of investigated samples (Table 5), a predominance of quercetin and naringenin content (54,474.4–66,677.2 and 13,769.1–16,093.0 µg/g, respectively) was observed in linseed samples. The main flavonoid compound in sesame samples appears to be naringenin (5543.4–7632.8 µg/g), followed by epicatechine (2097.6–3847.8 µg/g). In all other samples which were characterized by high flavonoid content (walnut, chia, peanuts) as well as pecan and pistachio samples, the main flavonoid was catechin. Besides high catechin content, chia seeds were also rich in naringin (2317.0–3839.3 µg/g). Peanut samples were also characterized by high rutin content compared to other investigated samples (ca. 2000 µg/g and higher). Though walnut samples did not have the highest overall flavonoid content, besides high catechin content, they were characterized by substantial contents of epicatechin (1065.6–1688.4 µg/g), naringenin (1064.4–1744.8 µg/g) and rutin (1651.8–2593.5 µg/g). In all investigated samples, except hemp seed samples, catechin and quercetin were determined. Unlike the values of catechin content, which fluctuate significantly, the content of quercetin in all examined samples was very similar, excluding linseed. Already mentioned hemp seed samples had the lowest flavonoid content (963.8–1093.1 µg/g).

**Figure 2:**
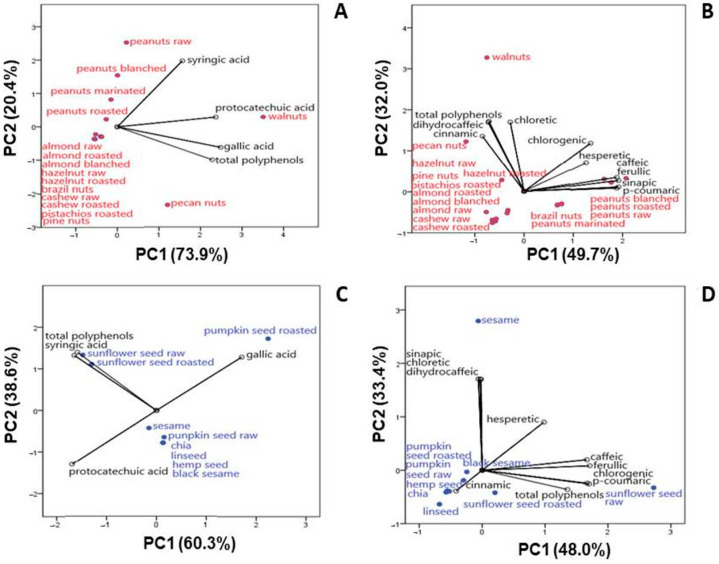
The biplot of PC1 and PC2 of HBAs and total phenolic content in nuts (**A**) and seeds (**C**); the biplot of PC1 and PC2 of HCAs and total phenolic content in nuts (**B**) and seeds (**D**).

Principal component analysis (PCA) was used to integrate the results of chemical parameters, discover the possible correlations among measured parameters, and classify the parameters in a factor plane. The analyzed HBAs and HCAs in nut and seed native samples were used to generate the PCA model. The contents of protocatechuic acid, gallic, syringic acid (as HBAs) and total phenolic content (TPC), as well as cinnamic, *p*-coumaric, caffeic, ferulic, isoferulic, synapic, dihydrocaffeic, phloretic, and chlorogenic acids (as HCAs) and TPC, were used as variables (Figure 2). The adequacy of the data for factor analysis was tested using the Kaiser–Meyer–Olkin test (KMO = 0.75) and Bartlett’s test for sphericity (*p* < 0.001). The number of components was chosen with the ordinary rule of selecting eigenvalues > 1.

PCA for HBAs in nuts showed that the first two eigenvalues of the correlation matrix accounted for 73.9% (PC1) and 20.4% (PC2) of the total variance of the data set (Figure 2A). Most of the native samples belonged to the central group, implying a similar elemental profile and making it difficult to distinguish the contribution of individual variables. According to PCA, walnuts, pecan nuts and all peanut samples were clearly separated from all other samples, indicating different HBAs. Walnuts were the most significant source of protocatechuic acid (strong positive correlation with the first axis: 0.923). Walnuts and pecan nuts were also the most significant sources of gallic acid and total phenolics (strong positive correlation with the first axis: 0.969 and 0.891, respectively), while raw peanuts were the most significant source of syringic acid (strong positive correlation with the second axis: 0.772). From the analyzed nut native samples (Table 2), walnut stands out as the richest source of HBAs tested. The predominance of walnut samples compared to other samples is due to their high protocatechuic and gallic acid contents (Table 3). Along with pecan nuts, they were the main sources of gallic acid and total phenolics, while raw peanuts and walnuts were the main sources of syringic acid.

PCA for HCAs in native nut samples showed that the first two eigenvalues of the correlation matrix accounted for 49.7% (PC1) and 32.0% (PC2) of the total variance of the data set (Figure 2B). Nut samples analyzed for HCA content show a difference in their composition. All native peanut samples (raw, marinated, blanched and roasted) were the most significant source of *p*-coumaric, ferulic and caffeic acids (a strong positive correlation with the first axis can be observed: 0.964, 0.963 and 0.947, respectively), while Brazil nuts were the most significant source of synapic acid (positive correlation with the first axis: 0.944). Walnuts and pecan nuts were also the most significant sources of dihydrocaffeic and phloretic acids, as well as TPC (a strong positive correlation with the second axis can be observed: 0.864, 0.855 and 0.850, respectively). The obtained values for the content of phenolics from the HCA group (Table 3) show that walnuts, pecan nuts and all peanuts stand out, while for TPC, the highest values are found in walnuts and pecan nuts (Table 1).

PCA for HBAs in native seed samples showed that the first two eigenvalues of the correlation matrix accounted for 60.3% (PC1) and 38.6% (PC2) of the total variance of the data set (Figure 2C). Seed samples analyzed for HBA content, similar to nut samples, show a difference in their composition. Raw sunflower seed and roasted sunflower seed samples were the most significant sources of syringic acid and total phenolics (a negative correlation with the first axis can be observed: −0.771 and −0.742, respectively). Roasted pumpkin seeds were the most significant source of gallic acid (positive correlation with the first axis: 0.800), while chia was the most significant source of protocatechuic acid (a negative correlation with the first axis: −0.792). In relation to the examined phenolics from the group of HBAs, the quantities listed in Table 3 show that chia seeds are the richest source of protocatechuic acid, roasted pumpkin seed is the richest source of gallic acid, and raw sunflower seed is the richest source of syringic acid and total phenolics.

In Figure 2D, the first axis has an overall variability of 48.0% (PC1) and the second axis has one of 33.4% (PC2), indicating that the selected variables were well represented by the PCA model. Raw sunflower seeds were the most significant source of ferulic, *p*-coumaric and caffeic acids, as well as total phenolics (strong positive correlation with the first axis: 0.989, 0.965, 0.963 and 0.784, respectively), while roasted sunflower seeds were the most significant source of chlorogenic acid (strong positive correlation with the first axis: 0.983). At the same time, sesame was revealed as the richest source of phloretic, dihydrocaffeic and synapic acids (strong positive correlation with the second axis: 0.988, 0.988 and 0.984, respectively) (Table 4).

The relationship between HBAs and TPC, as well as HCAs and TPC in nut and seed native samples, was investigated using the Pearson linear correlation coefficient (Figures S1 and S2).

Positive and significant correlations were observed between total phenolics and syringic acid in seed samples (r = 0.87, Figure S1B), and total phenolics and gallic acid in nut samples (r = 0.84, Figure S1A). However, in the nut samples, the strongest correlation was between acids belonging to HBAs, more precisely between gallic acid and protocatechuic acid (r = 0.93, Figure S1A).

A strong correlation was found between TPC and individual acids belonging to the group of HCAs (Figure S2). Seed samples also showed stronger correlations within and between HCAs and TPC than nut samples (Figure S2A,B).

Regarding the correlation between HCAs and total phenolics in nut samples, a strong correlation with total phenolics was confirmed with dihydrocaffeic acid (r = 0.84, Figure S2A). Seed samples showed stronger correlations within and between HCAs and phenolics than nut samples. In seeds it was between *p*-coumaric and ferulic acid (r = 0.96, Figure S2B), while in nuts there was a strong correlation between *p*-coumaric and caffeic acid (r = 0.87, Figure S2A). A strong correlation of total phenolics and chlorogenic acid was observed in seed samples (r = 0.93, Figure S2B).

## 4. Discussion

Both nuts and seeds are considered nutrient-dense foods due to their dietary fiber content and favorable composition and ratio of polyunsaturated to saturated fatty acids, all of which contribute to their highly praised health effects [14,26]. Nuts and seeds are somewhat different and are usually not considered together [27], though many findings highlight their comparable and complementary composition and content of certain nutrients (amino acids, omega-3 fatty acids, dietary fibers, and minerals), reinforcing their status as an indispensable part of a plant-based diet [3,28].

Besides their indisputable nutritional and economic importance, various nuts and seeds and their different by-products have recently been recognized as important sources of bioactive components [1]. Among these, phenolic compounds are the most studied in terms of providing positive health effects, particularly in the prevention of common chronic diseases such as diabetes, obesity, and hypertension [13,29]. Phenolic acids are recognized as dietary antioxidants that are readily absorbed in the human intestine. They also promote the anti-inflammatory capacity of humans and have protective effects against non-communicable diseases such as cardiovascular disease, cancer, diabetes and metabolic syndrome [12]. Some fruit wines showed the ability to increase the activity of enzymes of antioxidant protection (SOD, CAT and GPx) and decrease MDA content in isolated rat synaptosomes. This activity originates from phenolic acids (hydroxybenzoic and hydroxycinnamic derivatives) due to their significant ability to prevent oxidative stress damage [30].

As stated earlier, HCAs are present in many fruits and vegetables, thus contributing to a total phenolic content significantly greater than derivatives of benzoic acid [31]. Our results confirm this fact: in general, investigated native and defatted nut and seed extracts were richer sources of HCAs compared to HBAs (Table 2).

The results of our study also brought analyzed walnut and pecan samples to the fore, as well as samples of sunflower seeds and peanuts. This is mostly in line with the results of earlier research [1,8,14,16,32,33,34,35,36].

In the present study, walnut M and N samples had the highest TPC (153.33 and 150.11 µg GA/mg), followed by the pecan M sample (140.1 µg GA/mg). This is in accordance with a study of 11 different nuts obtained from Austrian local markets. Walnut samples had the highest TPC values (1020–2052 mg GAE/100g) followed by pecan (1022–1444 mg GAE/100g) [32]. The TPC of a Hungarian walnut cultivar had somewhat higher values (26.06 mg GAE/g) [37]. In two studies on different nuts from a Polish market [1,35], walnut samples were also the samples characterized by the highest phenolic content. In a study by Wojdylo et al. [1], flavonoid content was 432.9 mg/100g, comprising mostly flavanols (415.1 mg/100g), and no phenolic acids were detected. Walnut flavonoid content obtained in a study by Wozniak et al. [35] was substantially lower (114.861 µg/g), with predominance of epicatechine (114.296 µg/g). At the same time, phenolic acid content was 8.909 µg/g, with the main acids being caffeic and ferulic, and no HBAs were detected. These results are only partially corroborated by the findings of our study. Walnut samples were indeed characterized by high catechin (5927.4–6530.4 µg/g), epicatechin (1065.6–1688.4 µg/g) and overall flavonoid contents. Quite opposite to the previously mentioned investigations, the walnut samples in the present study were the main sources of HBAs (protocatechuic and gallic acid) with a content 2–3-fold higher compared to HCAs. The main HCAs in walnut samples in our study were dihydrocaffeic and phloretic acids (2477.6–3294.3 and 981.6–1059.9 µg/g).

Walnuts are usually consumed fresh, dried or in processed form, whole or ground, alone or with other edible nuts, honey and sweets. Regular consumption of walnut kernels or walnut oil has positive effects on immunity, memory and brain function, and dental health, reduces the risk of type II diabetes, and prevents and alleviates prostate cancer [38]. It also improves endothelial function by increasing endothelium-dependent vasodilatation (in hypercholesterolemic subjects) and helps maintain favorable cholesterol levels. Accordingly, the EFSA approved the health claim that “30 g of walnuts daily supports cardiovascular health and functions” [39]. The majority of evidence indicates that this is due to the well-balanced ratio of unsaturated fatty acids, but the influence of phenolic compounds should not be neglected. This also stands for major walnut phenolic acids. Gallic acid, known for its astringent and styptic effects, is also reported to have bacteriostatic, antineoplastic, antimelanogenic and antioxidant properties. Similarly, protocatechuic acid exhibits antioxidant, antimicrobial, cytotoxic, apoptotic and neuroprotective properties, and also inhibits LDL oxidation [40]. Phloretic acid was shown to reduce inflammation in dextran sulfate sodium (DSS)-induced colitis in mice, and it prevents plasma lipid accumulation and foam cell formation, thus decreasing the development of atherosclerosis [41]. It is produced in the colon as one of the most abundant products of colonic microbial catabolism of various (poly)phenols, reaching µM concentrations. It is usually better absorbed than its precursor molecules and has potential to contribute to health benefits associated with regular intake of a polyphenol-rich diet via direct radical quenching [42].

Pecan nuts also stood out as a significant source of various phenolics. In our study, pecan showed extremely high TPC (106.0–147.9 μg GA/mg). Native and defatted pecan samples also contained significant amounts of phenolic acids, especially HBAs, and flavonoids. The main phenolic acids were gallic and dihydrocaffeic acids. It was previously also concluded that pecans are excellent sources of flavonoids and condensed tannins (814 and 348 mg CE/100 g, respectively). At the same time, this amount of hydrophilic polyphenols was higher compared to walnut, macadamia and peanuts [14]. Accordingly, the pecan cakes also showed high flavonoid and tannin contents, and subsequent high antioxidant activity [8,10]. High inhibitory activity exhibited by pecan nuts towards α-amylase (>60%), pancreatic lipase (69%) and especially α-glucosidase (99%) implies their antidiabetic and anti-obesity activity [1].

Sunflower seeds were established among the richest sources of phenolic compounds in our study (Table 1 and Table 2). The main phenolic classes in our investigated sunflower seed samples were phenolic acids, with chlorogenic acid prevailing, while within the flavonoid class, quercetin and rutin dominated. The content of chlorogenic acid, the dominant compound in all sunflower seed samples, was much higher in defatted compared to native samples (Table 4). A similar phenolic profile was also obtained in the kernels of several sunflower genotypes grown in different locations in Turkey. TPC values ranged from 99.64 to 130.75 mg GAE/100 g, while TFC ranged from 838.33 to 1455.67 mg QE/100 g. The main flavonoids were quercetin (1.18–28.72 mg/100 g) and rutin (0.97–6.98 mg/100 g) [43]. Another study dealing with different sunflower cultivars revealed chlorogenic acid as the main phenolic acid in kernels (12.63–18.68 mg/g d.m.). Other marked phenolics were caffeic acid, ferulic acid and rosmarinic acid, along with myricetin and rutin, but they all were present in quantities lower than 0.15 mg/g d.m. Among many biological features, chlorogenic acid is a well-known antioxidant and its presence in sunflower seeds and kernels along with tocopherols provides substantial antioxidative protection [44]. Biological properties of chlorogenic acid, such as anti-inflammatory, anti-microbial, anti-obesity and antithrombotic properties, are also well known [45].

Peanut, a multipurpose oil-seed legume and major global oil-seed crop, has important nutritional and health significance [46]. According to some authors, the health benefits of peanut consumption are related to the bioactive components in the oil fraction. Nevertheless, many studies have shown that peanuts are a rich source of polyphenolic antioxidant compounds, including resveratrol, tocopherol, phytosterols, catechin, epicatechin, and quercetin, which were found to reduce neurodegenerative disease, hypertension, and cardiovascular disorders. Gallic acid (1.36–2.85 mg/L), protocatechuic (1.73–3.56 mg/L) and (-)-catechin (2.17–5.15 mg/L) were previously recognized as the key phenolic compounds of peanut kernels. Maturity stage and harvest time were identified to have a profound impact on the content of individual components, as well as on antioxidant activity [47]. In our study peanut samples were exceptionally rich in flavonoids. Compared to other samples, peanuts, along with walnuts, were the main sources of catechin and were characterized by a high content of rutin, quercetin and certain phenolic acids (gallic and protocatechuic). Among all peanut samples investigated, marinated and boiled ones had slightly lower values, though these differences were mostly not statistically significant. When TPC values are compared, differences are more pronounced (Table 1). This effect of processing in peanuts is already known. Zhang et al. [48] showed that roasting has lesser effects on fatty acids and free amino acids in peanuts and induces relatively small effects on nutritional components compared with boiling, being an ideal processing method for peanuts. This was also shown for antioxidant activity: boiling substantially reduced the TPC and antioxidant capacity of peanuts. On the contrary, frying and baking had a significant promoting effect on TPC [49]. This could be explained by the fact that raw and roasted peanut samples were with skin, while boiled and marinated samples were not. Namely, as previously shown [50], monomeric flavonoids (catechin and epicatechin) and procyanidins are the main phenolics in peanut skins, while phenolic acids are characteristic of kernels. Furthermore, high roasting temperatures cause degradation of condensed and hydrolysable tannins [51] and also the release of bound phenolic acids [52].

Chia seed samples also emerged as an exceptionally rich flavonoid source, especially of catechin (3749.6–6061.4 µg/g), while the main HCAs were hesperetic (532.5–614.3 µg/g), phloretic (540.0–1033.8 µg/g) and chlorogenic acids (417.1–564.7 µg/g). In previous research, caffeic (27–30 µg/g) and chlorogenic acid (4.68 µg/g), quercetin, myricetin and kaempferol were reported as the main phenolics in chia seeds [53,54]. The health-promoting potential of chia seeds is recognized based on their strong nutritional profile, as well as the presence of various bioactive compounds including phenolics [55].

On the other hand, some commonly and frequently consumed nuts and seeds had very low TPC. This primarily applies to almond and pine nut samples (Table 1). According to previous research, no phenolics were detected in pine nuts, while the phenolic acid content in almonds was only 3 μg/g. Almond and pine nuts showed the lowest potential towards pancreatic lipase, but interestingly, the almond sample inhibited α-glucosidase by 93% [1]. According to our results, the TPC in almond and pine nut samples was not negligible (3.1–6.8 and 5.3 µg GA/mg, respectively), but still was significantly lower compared to other analyzed nut and seed samples (Table 2). These findings are also consistent with the results of some earlier studies. Pine nuts are known for their low phenolic content (32 mg GAE/100 g) followed by almonds without skin (47 mg GAE/100 g) [32]. In almond seed cakes, TPC previously could not be determined [8]. Low TPC (below 10 µg GA/mg) was characteristic of native pumpkin seeds which are consumed for their taste and health benefits. They were almost devoid of flavonoids (<1 µg GA/mg). Still, these samples, especially defatted ones, were rich sources of gallic acid (ca. 1500 µg/g and more). The main flavonoids of pumpkin seed samples were catechin and quercetin. Hemp seeds were also characterized by low TPC (ca. 10 µg/g) and TFC (<1 µg GA/mg), with gallic acid as the main phenolic acid and naringenin and epicatechine dominant in the flavonoid fraction. Previously, naringenin, syringic and benzoic acids were identified as the main phenolics in hemp seeds. The amount of biologically active compounds also varied, with TPC ranging from 6.55 to 12.39 mg/g RUE and TFC ranging from 2.52 to 4.74 mg/g RUE [56].

Esculin, coumarin glycoside, is typically present in many plants of genus *Fraxinus*, chestnut flowers and seeds, as well as in cassava roots (*Manihot esculenta*). It demonstrates various biological effects, including antioxidant, anti-inflammatory, neuroprotective and anti-diabetic, making it potentially pleotropic agent in the treatment or prevention of non-communicable diseases (NCDs) [57,58]. Within our study esculin was detected in very low concentrations in the majority of samples (Table 2). The highest esculin content was observed in chia seed samples (2065.4–3238.7 µg/g). In chia seeds esculin was previously detected along with another coumarin, daphnetin [59]. The presence of these coumarins was also shown in peanuts: red ones contain higher amounts of both aforementioned coumarins compared to pink or white-colored ones [60].

Besides the abovementioned nuts and seeds, characterized by the highest or the lowest phenolic contents, our investigation comprised other nuts and seeds that are commonly used (hazelnuts, cashew, pistachios, Brazil nuts, sesame, linseed). Though these samples in general did not exhibit extremely high TPC values, the results of their phenolic content and profile significantly substantiate the existing data on their composition.

This work represents the first comprehensive analysis of phenolic components of the most commonly consumed nuts and seeds from the Serbian market. The methodology of extraction applied in this work aimed to obtain as many homogenous results as possible considering natural differences between the samples investigated. In that way all the samples were treated the same and could be compared directly, since they are consumed the same way. Compared to the literature, our results on individual samples are higher and the compositions of individual phenolics are somewhat different. As stated earlier, literature data are very heterogenous, considering origin of the samples, processing and methodology used, making comparison of the results very difficult and sometimes impossible. Nevertheless, taking into account all possible limitations and obstacles, according to our results, walnut, pecan, sunflower seeds and peanuts emerged as the richest sources of various phenolics, highly correlating with findings of many previous studies [8,9,10].

Besides this, a particularly important aspect of the present study is the concurrent analysis of the native and defatted nuts and seeds. For most of the analyzed defatted samples, the phenolic content and profile were comparable with their native counterparts, but in some cases, phenolic content in defatted samples was higher (sunflower seeds, pumpkin seed, chia; Table 1). Published research supports the technological and nutritional relevance of by-products derived from pumpkin, sunflower, flaxseed, sesame, chia seeds and peanuts identified in our study as rich sources of HCAs, HBAs and flavonoids. Pumpkin seeds are valued for their health benefits and are also an important source of highly appreciated edible oil. Similarly, considering the amount of seedcake remaining after oil production and its nutritional and phenolic composition, pumpkin seed flour is also a very important functional food component. Pumpkin seed oil cake has been successfully used in gluten-free bread, improving nutritional, technological and sensory attributes [61,62]. Samples of defatted raw and roasted sunflower seeds in our study were characterized by much higher TPC than native ones and were the richest sources of HCAs, especially chlorogenic acid (ca. 100 mg/g and higher; Table 2). This fact could be particularly significant if the amount of sunflower press cake (SPC), the main by-product of the sunflower oil industry, is taken into account. SPC has a rich nutritional composition and is mainly used as high-quality livestock feed and organic fertilizer, but is considered as a new raw material with innovative applications, particularly as a sustainable source of plant-based proteins [63]. Our results additionally establish defatted sunflower products as important natural sources of phenolic compounds, especially chlorogenic acid. Though positive biological effects of chlorogenic acid are known and desirable in functional food products, it still shows some unwanted effects: it interacts with proteins and changes their solubility, digestibility, shelf life and stability, as well as organoleptic properties. Thus, by-products of sunflower seeds could be considered as important sources of pure chlorogenic acid as well [45]. Flaxseed cake is well established as a nutrient-dense by-product, enriched in proteins, fibers, minerals and phenolics, and is widely proposed for fortifying cereal-based and protein-enriched foods [4]. Sesame oil cake is also recognized as a phenolic-rich material with notable antioxidant capacity and strong potential for circular economy valorization within food systems [18]. Chia seed oil extraction residues further expand the portfolio of edible by-products, providing a fiber- and protein-rich material suitable for bakery and snack applications [64]. These reports align with our results, which indicate that sunflower, sesame, pumpkin, flaxseed and chia samples exhibited the highest levels of key phenolic compounds, supporting their prioritization as realistic and scalable ingredients for functional food development. In addition, recent findings demonstrate that defatted peanut cake flour, an abundant and widely available by-product of peanut oil extraction, can enhance both the nutritional and functional quality of bakery products. Its incorporation increases total phenolic content and DPPH antioxidant activity while maintaining acceptable sensory properties up to 10% substitution, confirming its technological feasibility for fortification strategies in baked goods [65]. In contrast, although exceptionally rich in protocatechuic, gallic and other phenolic acids, walnuts and pecans are not typically processed into oil on an industrial scale and therefore do not generate substantial quantities of press cake. Their defatted fractions should therefore be regarded as high-value, phenolic-rich ingredients suitable for targeted nutritional and antioxidant enrichment, rather than as scalable by-products comparable to oilseed cakes.

The results also indicate the possibility of using both native and defatted samples in functional food products, as the differences in phenolic content between them are not substantial in most cases. By combining data on nutritional composition and the knowledge of the phenolic profile of respective nuts and seeds, it would be possible to formulate a product that optimizes nutritive, functional and organoleptic properties.

## 5. Conclusions

In this work, the HPLC phenolic profile, including phenolic acids, flavonoids and coumarins, in native and defatted samples of 25 commercial nuts and seeds from the Serbian market was analyzed. A comparison within samples was also performed in order to determine the significance of native samples, as well as their by-products, as potential sources of phenolics, with special emphasis on phenolic acids.

Of all the samples analyzed, sunflower seeds, especially the raw ones, proved to be an exceptionally rich source of chlorogenic acid, exceeding the values previously reported in the literature. Similarly, walnuts and pecans showed the highest total phenolic content, as well as the highest levels of protocatechuic and gallic acids, confirming their dominance in terms of phenolic acid content. In addition, the unexpectedly high flavonoid concentrations in peanut and chia seed samples suggest that these commonly consumed foods may have greater bioactive potential than previously thought.

The present research confirmed the fact that different nuts and seeds and their defatted by-products could be considered as valuable but still underutilized sources of various phenolics. Overall, as one of the most detailed studies on phenolic acid profiles of native and defatted nut and seed samples available on the Serbian market, it provides valuable baseline data for future research and development of functional foods and innovative strategies that can support sustainable food systems and the goals of the UN 2030 Agenda. Although the provided data confirmed the main findings of many previous studies, they highlighted some important and relatively constant characteristics of phenolic composition and content in certain nuts and seeds, i.e., walnuts, pecans and sunflower seeds. This enables focusing further research on the samples with the highest TPC, suggesting high biological potential. Knowing the chemical profile in terms of phenolic compounds is useful for future investigations towards developing products with a uniform and standardized composition, one of the prerequisites for high-quality products with pronounced activity. Future research should also encompass investigations on bioavailability and health effects in vivo in order to substantiate their role in the prevention of chronic diseases.

## Figures and Tables

**Table 1:** Total phenolic (TPC) and total flavonoid content (TFC).

Sample		TPC (µg GA/mg)	TFC (µg C/mg)
Peanuts raw	Native	46.3 ± 1.3	23.6 ± 4.3 ^a^
	Macerate	52.8 ± 1.5	22.7 ± 1.7 ^a^
	Soxhlet	46.2 ± 1.3	14.9 ± 1.6
Roasted peanuts	Native	52.1 ± 2.3 ^a,b^	12.4 ± 1.0
	Macerate	37.6 ± 1.2	12.9 ± 1.5
	Soxhlet	40.9 ± 1.7	11.1 ± 0.3
Boiled peanuts	Native	16.0 ± 0.5 ^a^	3.6 ± 0.4
	Macerate	17.8 ± 0.5	4.0 ± 1.3
	Soxhlet	18.9 ± 0.5	4.2 ± 0.2
Marinated peanuts	Native	15.5 ± 0.5 ^a,b^	2.68 ± 0.3 ^a^
	Macerate	17.7 ± 0.4 ^b^	4.9 ± 0.3 ^b^
	Soxhlet	13.9 ± 0.4	2.8 ± 0.3 ^a^
Raw almond	Native	6.8 ± 0.2 ^a,b^	6.8 ± 0.6 ^a,b^
	Macerate	2.9 ± 0.0 ^b^	2.7 ± 0.2 ^b^
	Soxhlet	3.9 ± 0.1	4.1 ± 0.8
Roasted almond	Native	5.9 ± 0.1 ^a,b^	3.8 ± 0.1
	Macerate	4.0 ± 0.0	4.3 ± 1.5
	Soxhlet	4.4 ± 0.0	3.8 ± 0.5
Boiled almond	Native	3.1 ± 0.1 ^a^	2.9 ± 0.7 ^a^
	Macerate	2.9 ± 0.1 ^a^	2.8 ± 0.3 ^a^
	Soxhlet	2.0 ± 0.1	2.1 ± 0.1
Raw hazelnuts	Native	18.2 ± 0.3	13.8 ± 0.1
	Macerate	28.1 ± 0.9 ^a^	13.8 ± 2.4
	Soxhlet	17.8 ± 0.7	11.0 ± 0.2
Roasted hazelnuts	Native	8.8 ± 0.1 ^a,b^	5.4 ± 0.7
	Macerate	7.7 ± 0.2	3.4 ± 0.9 ^a^
	Soxhlet	7.1 ± 0.2	5.3 ± 2.6
Walnut	Native	150.1 ± 2.4 ^a^	28.0 ± 1.7 ^a^
	Macerate	155.3 ± 5.7 ^a^	28.6 ± 5.0 ^a^
	Soxhlet	132.5 ± 2.2	83.6 ± 9.7
Brazil nut	Native	38.4 ± 0.9	12.6 ± 0.6 ^a,b^
	Macerate	26.4 ± 0.8 ^a^	4.74 ± 0.9 ^b^
	Soxhlet	35.3 ± 0.9	7.99 ± 1.73
Raw cashew	Native	9.6 ± 0.2	10.7 ± 1.4 ^a,b^
	Macerate	9.4 ± 0.1	5.5 ± 1.2 ^b^
	Soxhlet	8.7 ± 0.2	4.3 ± 0.9
Roasted cashew	Native	9.8 ± 0.3 ^a^	3.5 ± 0.2
	Macerate	9.8 ± 0.3 ^a^	3.6 ± 0.3
	Soxhlet	7.6 ± 0.1	4.0 ± 0.7
Pecan nut	Native	148.0 ± 4.7 ^a^	61.3 ± 18.0 ^a,b^
	Macerate	140.1 ± 4 ^a^	39.4 ± 3.8 ^b^
	Soxhlet	106.0 ± 2.9	50.5 ± 12.5
Pine nuts	Native	5.4 ± 0.1	1.4 ± 0.5 ^a,b^
	Macerate	5.5 ± 0.2	0.2 ± 0.1 ^b^
	Soxhlet	5.2 ± 0.4	0.4 ± 0.2
Roasted pistachios	Native	38.3 ± 1.0 ^a,b^	9.5 ± 1.1 ^a,b^
	Macerate	21.7 ± 0.6 ^b^	4.4 ± 0.2
	Soxhlet	18.8 ± 0.5	5.3 ± 1.1
Sesame	Native	12.9 ± 0.3	8.3 ± 2.1 ^a,b^
	Macerate	12.4 ± 0.3	4.1 ± 0.7 ^b^
	Soxhlet	12.0 ± 0.6	1.5 ± 0.7
Black sesame	Native	15.5 ± 0.7 ^a^	5.4 ± 0.7 ^a^
	Macerate	15.6 ± 1.0 ^a^	5.5 ± 1.8 ^a^
	Soxhlet	20.2 ± 1.1	9.1 ± 0.7
Raw sunflower seed	Native	93.3 ± 4.6 ^a,b^	103.3 ± 9.2 ^a,b^
	Macerate	129.1 ± 0.1 ^b^	157.4 ± 9.9
	Soxhlet	170.1 ± 4.1	147.1 ± 8.5
Roasted sunflower seed	Native	98.4 ± 7.0 ^a,b^	121.3 ± 15.2
	Macerate	115.0 ± 12.8 ^b^	101.7 ± 10.6 ^a^
	Soxhlet	132.0 ± 9.8	120.5 ± 13.7
Raw pumpkin seed	Native	9.6 ± 0.3 ^a,b^	0.9 ± 0.6
	Macerate	11.4 ± 0.3 ^b^	0.3 ± 0.2 ^a^
	Soxhlet	14.5 ± 0.5	0.8 ± 0.1
Roasted pumpkin seed	Native	9.4 ± 0.4	0.5 ± 0.4 ^a,b^
	Macerate	10.1 ± 0.2	0.3 ± 0.0 ^b^
	Soxhlet	10.8 ± 0.6	0.2 ± 0.1
Chia	Native	44.7 ± 1.3 ^a,b^	34.0 ± 1.6 ^a,b^
	Macerate	53.2 ± 3.6 ^b^	40.8 ± 4.8 ^b^
	Soxhlet	65.7 ± 0.9	54.8 ± 3.1
Linseed	Native	23.8 ± 0.7 ^a^	6.6 ± 0.3 ^a^
	Macerate	25.3 ± 0.6 ^a^	5.8 ± 0.3 ^a^
	Soxhlet	46.7 ± 2.0	10.1 ± 1.5
Hemp seed	Native	9.6 ± 0.2 ^a^	0.7 ± 0.1 ^a,b^
	Macerate	10.8 ± 0.7	0.2 ± 0.0 ^b^
	Soxhlet	11.7 ± 0.6	0.1 ± 0.0

GA—gallic acid; C—catechin; the values are represented as mean ± SD; means with different lowercase letters between various extracts indicate significant differences (*p* < 0.05).

**Table 2:** Content of hydroxybenzoic acids (HBAs), hydroxycinnamic acids (HCAs), flavonoids and coumarins (µg/g).

Sample		HBAs	HCAs	Flavonoids	Coumarins
Peanuts raw	Native	1107.5 ± 162.1 ^a^	4397.2 ± 350.1 ^a^	12,184.5 ± 2415.1 ^a^	151.0 ± 12.7 ^a,b^
	Macerate	771.3 ± 4.8 ^b^	2861.7 ± 220.3 ^b^	9434.9 ± 1926.2 ^b^	209.6 ± 115.3 ^b^
	Soxhlet	1104.1 ± 150.7	4291.9 ± 376.4 ^a^	12,802.5 ± 2834.1 ^a^	58.0 ± 8.5
Roasted peanuts	Native	835.9 ± 123.7 ^a^	3766.3 ± 281.5 ^a,b^	9644.2 ± 1968.8 ^a,b^	90.9 ± 35.4 ^a^
	Macerate	982.0 ± 80.0	4315.6 ± 401.8 ^b^	12,637.0 ± 2980.6 ^b^	90.3 ± 17.0 ^a^
	Soxhlet	971.4 ± 81.3	4969.1 ± 407.1	11,713.1 ± 2711.4	115.9 ± 2.8
Boiled peanuts	Native	987.3 ± 14.6 ^a^	3623.8 ± 264.3 ^a,b^	11,049.0 ± 2347.7	144.8 ± 20.5 ^a^
	Macerate	861.2 ± 20.7 ^b^	3858.6 ± 317.7	10,733.2 ± 2135.0	135.5 ± 7.8 ^a^
	Soxhlet	964.8 ± 13.1 ^a^	4010.6 ± 293.7	12,208.3 ± 2600.7	187.9 ± 29.7
Marinated peanuts	Native	863.6 ± 11.8 ^a,b^	3261.0 ± 289.8 ^a^	9400.4 ± 1949.5 ^a^	130.8 ± 26.9 ^a,b^
	Macerate	756.2 ± 72.0	2973.3 ± 247.3 ^b^	7664.4 ± 1301.9 ^b^	76.1 ± 28.3 ^b^
	Soxhlet	788.5 ± 37.9	3128.7 ± 281.6 ^a^	9219.4 ± 1662.3	83.0 ± 41.0
Raw almond	Native	706.7 ± 211.0 ^a,b^	974.8 ± 109.8 ^a,b^	2564.15 ± 540.9 ^a^	<LOQ
	Macerate	579.1 ± 185.6	1083.1 ± 174.5 ^b^	1938.45 ± 379.5 ^b^	<LOQ
	Soxhlet	603.9 ± 173.7	1475.4 ± 228.1	2472.43 ± 483.0 ^a^	<LOQ
Roasted almond	Native	741.2 ± 214.0 ^a,b^	1141.4 ± 169.4 ^a,b^	2706.05 ± 519.5 ^a,b^	<LOQ ^a^
	Macerate	684.9 ± 186.2 ^b^	1540.8 ± 264.6 ^b^	2473.67 ± 471.4 ^b^	13.4 ± 3.5 ^b^
	Soxhlet	649.4 ± 177.0	1292.7 ± 228.0	2258.43 ± 450.6	<LOQ ^a^
Boiled almond	Native	579.1 ± 168.3 ^a^	752.7 ± 104.0	1684.285 ± 477.5 ^a,b^	<LOQ ^a^
	Macerate	637.0 ± 172.3 ^b^	783.4 ± 124.6	1912.7 ± 570.3	<LOQ ^a^
	Soxhlet	587.5 ± 173.5 ^a^	801.8 ± 128.7	1864.92 ± 512.6	24.8 ± 33.9
Raw hazelnuts	Native	629.8 ± 183.2 ^a^	2766.9 ± 488.1 ^a,b^	3684.49 ± 430.2 ^a^	<LOQ
	Macerate	797.9 ± 233.0 ^b^	4125.2 ± 602.7	4549.7 ± 488.8 ^b^	<LOQ
	Soxhlet	544.3 ± 159.7 ^a^	3634.9 ± 613.8	4082.9 ± 548.1 ^b^	<LOQ
Roasted hazelnuts	Native	596.1 ± 198.4 ^a^	1893.0 ± 516.3 ^a,b^	4945.9 ± 512.1 ^a^	31.9 ± 4.0 ^a,b^
	Macerate	539.1 ± 167.9	1648.0 ± 190.5 ^b^	4224.2 ± 451.4	43.4 ± 7.8
	Soxhlet	482.8 ± 151.8	1340.0 ± 153.3	4363.4 ± 567.0	43.8 ± 9.9
Walnut	Native	9717.2 ± 292.8 ^a,b^	5318.4 ± 980.9 ^a^	13,844.5 ± 280.7 ^a^	112.3 ± 13.4 ^a^
	Macerate	15,809.5 ± 509.0 ^b^	5466.5 ± 1052.4 ^a^	14,618.1 ± 285.0 ^a^	151.1 ± 42.4 ^a^
	Soxhlet	11,445.4 ± 914.4	4591.2 ± 799.8	11,611.8 ± 1995.4	119.2 ± 62.9
Brazil nut	Native	944.1 ± 281.3 ^a,b^	3560.9 ± 388.0 ^a^	4093.0 ± 655.3 ^a,b^	24.5 ± 5.0 ^a,b^
	Macerate	863.3 ± 249.3 ^b^	3610.0 ± 518.3 ^a^	3002.8 ± 398.6	52.7 ± 1.4
	Soxhlet	735.2 ± 241.7	4043.4 ± 601.8	3175.3 ± 423.0	56.4 ± 4.2
Raw cashew	Native	650.2 ± 243.8 ^a^	1463.9 ± 249.7 ^a^	2800.0 ± 452.7 ^a,b^	<LOQ ^a^
	Macerate	507.1 ± 170.7 ^a^	1479.3 ± 275.4 ^a^	2323.6 ± 353.3 ^b^	11.5 ± 3.5 ^b^
	Soxhlet	653.5 ± 241.4	2352.2 ± 351.9	3227.6 ± 495.5	<LOQ ^a^
Roasted cashew	Native	625.9 ± 205.9 ^a^	1895.9 ± 285.7 ^a,b^	2135.1 ± 399.3 ^a^	<LOQ ^a^
	Macerate	614.3 ± 201.0 ^a^	2299.0 ± 381.7	2326.2 ± 396.5	16.5 ± 2.0 ^b^
	Soxhlet	579.6 ± 187.0	2457.3 ± 263.3	2467.4 ± 428.2	<LOQ ^a^
Pecan nut	Native	1777.1 ± 153.7	3354.0 ± 827.5 ^a,b^	6430.7 ± 769.0 ^a^	<LOQ
	Macerate	1714.2 ± 657.5	3894.8 ± 995.6	7318.4 ± 964.8 ^b^	<LOQ
	Soxhlet	1762.5 ± 668.9	3636.5 ± 915.1	6603.2 ± 816.5 ^a^	<LOQ
Pine nuts	Native	390.9 ± 161.6 ^a^	273.4 ± 76.1 ^a^	2932.9 ± 570.1 ^a,b^	<LOQ
	Macerate	495.4 ± 188.4 ^b^	252.7 ± 72.1 ^a^	3651.8 ± 703.1	<LOQ
	Soxhlet	409.1 ± 176.1 ^a^	229.3 ± 66.1	3421.1 ± 708.2	<LOQ
Roasted pistachios	Native	1019.7 ± 362.6 ^a^	1336.4 ± 160.6	5166.2 ± 729.2 ^a^	<LOQ
	Macerate	1575.5 ± 581.3 ^b^	1239.4 ± 157.4	5209.5 ± 722.3 ^a^	<LOQ
	Soxhlet	1081.8 ± 362.0 ^a^	1232.7 ± 162.4	6220.1 ± 796.9	<LOQ
Sesame	Native	760.7 ± 226.2 ^a^	6075.2 ± 657.8 ^a,b^	13,806.7 ± 995.3	101.4 ± 3.5
	Macerate	829.7 ± 259.9 ^b^	4661.4 ± 622.9	15,451.8 ± 567.6	107.2 ± 10.6
	Soxhlet	746.8 ± 233.4 ^a^	4626.1 ± 694.2	13,783.6 ± 677.4	95.5 ± 5.0
Black sesame	Native	553.1 ± 182.0 ^a,b^	1841.0 ± 182.9 ^a,b^	4504.7 ± 558.5 ^a^	57.7 ± 2.8 ^a,b^
	Macerate	732.8 ± 244.9	2130.0 ± 188.9	4977.7 ± 605.3 ^b^	72.5 ± 3.5
	Soxhlet	624.4 ± 216.8	2035.8 ± 179.6	4313.4 ± 483.5 ^a^	72.0 ± 5.7
Raw sunflower seed	Native	2386.4 ± 483.9 ^a,b^	120,429.6 ± 832.3 ^a,b^	4231.0 ± 599.5 ^a,b^	117.3 ± 7.1 ^a,b^
	Macerate	1665.1 ± 244.3 ^b^	136,137.4 ± 440.9	4517.2 ± 577.0	81.4 ± 2.1 ^b^
	Soxhlet	1487.5 ± 224.3	137,230.6 ± 334.6	4492.7 ± 640.1	97.9 ± 7.1
Roasted sunflower seed	Native	1525.8 ± 188.3 ^a,b^	89,629.7 ± 259.1 ^a,b^	4182.7 ± 660.2	53.7 ± 43.8 ^a,b^
	Macerate	1213.4 ± 237.0	102,079.7 ± 388.2	4201.2 ± 597.4	83.0 ± 10.6 ^b^
	Soxhlet	1220.1 ± 320.2	107,533.1 ± 352.1	4219.9 ± 585.1	74.5 ± 10.6
Raw pumpkin seed	Native	948.5 ± 515.2 ^a,b^	1057.1 ± 145.8 ^a,b^	2480.8 ± 403.8 ^a,b^	82.4 ± 1.4 ^a,b^
	Macerate	1664.8 ± 903.1	1293.7 ± 214.9 ^b^	3297.6 ± 556.0	143.7 ± 24.8 ^b^
	Soxhlet	1703.5 ± 918.5	1569.3 ± 267.6	3365.8 ± 592.3	104.3 ± 9.9
Roasted pumpkin seed	Native	1169.5 ± 157.9 ^a,b^	988.5 ± 128.5 ^a^	2851.9 ± 478.7 ^a^	62.0 ± 7.1 ^a,b^
	Macerate	1761.1 ± 268.9	1357.2 ± 199.0 ^b^	3159.5 ± 485.9 ^b^	96.8 ± 12.0
	Soxhlet	1603.7 ± 303.8	1000.8 ± 160.1 ^a^	2988.0 ± 480.3 ^a^	93.9 ± 3.5
Chia	Native	927.7 ± 304.9 ^a,b^	2096.1 ± 213.0 ^a^	10,754.9 ± 1567.4 ^a,b^	2065.4 ± 182.4 ^a,b^
	Macerate	1013.1 ± 350.6 ^b^	2602.0 ± 348.2 ^b^	9714.7 ± 1495.3 ^b^	2299.0 ± 106.1 ^b^
	Soxhlet	1133.5 ± 428.7	2191.1 ± 261.8 ^a^	13,012.0 ± 2323.9	3238.7 ± 176.1
Linseed	Native	286.9 ± 165.6 ^a,b^	4300.5 ± 949.2 ^a,b^	79,880.3 ± 2226.6 ^a,b^	65.5 ± 5.0 ^a,b^
	Macerate	402.1 ± 232.1	3984.3 ± 809.7	71,882.6 ± 2190.8 ^b^	73.1 ± 0.7 ^b^
	Soxhlet	436.5 ± 252.0	3953.6 ± 761.4	83,382.3 ± 2379.4	80.4 ± 5.0
Hemp seed	Native	959.1 ± 341.4 ^a,b^	1656.8 ± 195.6 ^a,b^	1093.1 ± 222.1 ^a,b^	741.9 ± 8.5 ^a,b^
	Macerate	1218.5 ± 477.1 ^b^	2034.8 ± 265.3 ^b^	963.8 ± 194.7	948.2 ± 36.8
	Soxhlet	612.6 ± 178.8	1831.7 ± 247.1	1011.3 ± 203.4	979.5 ± 24.8

LOQ: limit of quantification; the values are represented as mean ± SD; means with different lowercase letters between various extracts indicate significant differences (*p* < 0.05).

**Table 3:** Hydroxybenzoic acid (HBA) content (µg/g).

Sample		Protocatechuic Acid	Gallic Acid	Syringic Acid
Peanuts raw	Native	283.5 ± 1.2	267.9 ± 2.8	556.1 ± 25.4 ^a^
	Macerate	251.7 ± 0.0	260.8 ± 1.9	258.8 ± 9.3 ^b^
	Soxhlet	286.5 ± 20.0	275.7 ± 3.8	542.0 ± 39.3 ^a^
Roasted peanuts	Native	398.6 ± 4.6	285.8 ± 8.1	151.6 ± 25.2 ^a.b^
	Macerate	339.9 ± 6.3 ^a^	241.7 ± 5.1	400.3 ± 18.9 ^b^
	Soxhlet	414.6 ± 21.3	299.2 ± 3.5	257.7 ± 5.6
Boiled peanuts	Native	322.1 ± 9.1	319.3 ± 8.7	345.9 ± 11.4 ^a^
	Macerate	289.4 ± 25.5	306.5 ± 11.1	265.3 ± 16.3 ^b^
	Soxhlet	307.7 ± 6.9	323.5 ± 19.2	333.7 ± 16.8 ^a^
Marinated peanuts	Native	300.1 ± 11.6	276.5 ± 0.5	287.1 ± 47.9
	Macerate	266.4 ± 23.1	315.8 ± 8.9	174.0 ± 90.5
	Soxhlet	281.7 ± 8.1	287.5 ± 38.5	219.2 ± 77.4
Raw almond	Native	299.3 ± 9.6 ^a,b^	407.4 ± 21.1 ^a^	<LOQ ^a^
	Macerate	208.9 ± 24.8	370.2 ± 19.5	<LOQ ^a^
	Soxhlet	243.5 ± 39.1	350.0 ± 25.4	10.4 ± 5.5
Roasted almond	Native	369.4 ± 16.0	371.8 ± 23.5	<LOQ ^a,b^
	Macerate	302.6 ± 18.2	365.9 ± 9.2	16.4 ± 2.9
	Soxhlet	296.5 ± 3.7	339.3 ± 2.7	13.6 ± 3.0
Boiled almond	Native	270.0 ± 0.8	309.2 ± 38.5	<LOQ^a^
	Macerate	276.7 ± 17.0	343.2 ± 21.1	17.2 ± 6.4 ^b^
	Soxhlet	257.0 ± 11.2	330.5 ± 18.8	<LOQ ^a^
Raw hazelnuts	Native	292.7 ± 15.8	337.2 ± 2.5 ^a^	<LOQ
	Macerate	363.8 ± 18.6 ^a^	434.1 ± 34.3 ^b^	<LOQ
	Soxhlet	243.7 ± 27.4	300.6 ± 21.4 ^a^	<LOQ
Roasted hazelnuts	Native	199.3 ± 11.5	396.7 ± 16.1 ^a^	<LOQ
	Macerate	206.5 ± 24.8	332.6 ± 13.7	<LOQ
	Soxhlet	181.3 ± 12.5	301.5 ± 14.7	<LOQ
Walnut	Native	6369.5 ± 502.8 ^a^	2844.1 ± 262.1 ^a,b^	503.6 ± 29.2 ^a^
	Macerate	11,551.8 ± 765.6 ^b^	3859.6 ± 346.2 ^b^	398.2 ± 68.9
	Soxhlet	5775.9 ± 8.2 ^a^	5203.3 ± 5.4	466.2 ± 1.7
Brazil nut	Native	541.9 ± 7.6 ^a,b^	402.2 ± 11.6 ^a^	<LOQ
	Macerate	425.5 ± 8.3 ^b^	437.8 ± 21.1	<LOQ
	Soxhlet	251.9 ± 5.2	483.3 ± 6.4	<LOQ
Raw cashew	Native	169.6 ± 9.3	480.6 ± 20.8	<LOQ ^a^
	Macerate	142.6 ± 10.6	351.3 ± 45.5 ^a^	13.1 ± 2.1 ^b^
	Soxhlet	176.1 ± 9.8	477.3 ± 1.9	<LOQ ^a^
Roasted cashew	Native	214.1 ± 16.5	411.8 ± 16.4	<LOQ
	Macerate	212.5 ± 2.3	401.8 ± 4.8	<LOQ
	Soxhlet	206.3 ± 4.9	373.3 ± 4.6	<LOQ
Pecan nut	Native	483.3 ± 7.8	1293.8 ± 18.0	<LOQ
	Macerate	424.2 ± 3.8	1290.0 ± 12.3	<LOQ
	Soxhlet	447.0 ± 15.9	1315.5 ± 15.7	<LOQ
Pine nuts	Native	<LOQ	311.1 ± 4.7	79.8 ± 2.9 ^a^
	Macerate	<LOQ	370.4 ± 6.0	125.1 ± 3.9 ^b^
	Soxhlet	<LOQ	335.2 ± 5.5	73.9 ± 8.4 ^a^
Roasted pistachios	Native	298.1 ± 1.7 ^a^	721.6 ± 17.2 ^a^	<LOQ
	Macerate	425.8 ± 5.9 ^b^	1149.8 ± 18.4 ^b^	<LOQ
	Soxhlet	357.9 ± 58.8 ^b^	723.9 ± 52.8 ^a^	<LOQ
Sesame	Native	287.8 ± 2.2	460.6 ± 6.8	12.2 ± 5.9 ^a,b^
	Macerate	313.8 ± 0.6	515.8 ± 1.7	<LOQ
	Soxhlet	284.0 ± 0.2	462.9 ± 21.2	<LOQ
Black sesame	Native	189.3 ± 8.5 ^a^	363.9 ± 5.0 ^a^	<LOQ
	Macerate	243.0 ± 13.2 ^b^	489.8 ± 20.8 ^b^	<LOQ
	Soxhlet	191.7 ± 7.9 ^a^	432.7 ± 14.5 ^b^	<LOQ
Raw sunflower seed	Native	302.5 ± 7.2	814.3 ± 15.0 ^a^	1269.6 ± 25.6 ^a,b^
	Macerate	340.1 ± 21.4	820.7 ± 16.1 ^a^	504.3 ± 16.8
	Soxhlet	316.3 ± 2.6	747.3 ± 19.6	423.9 ± 33.2
Roasted sunflower seed	Native	291.4 ± 21.9	609.7 ± 0.5 ^a^	624.7 ± 7.4 ^a,b^
	Macerate	318.0 ± 1.3	672.6 ± 38.0 ^a^	222.8 ± 32.3 ^b^
	Soxhlet	355.3 ± 49.1	749.5 ± 1.9	115.3 ± 5.3
Raw pumpkin seed	Native	<LOQ	910.7 ± 17.7 ^a,b^	37.8 ± 7.2 ^a,b^
	Macerate	<LOQ	1597.1 ± 6.9	67.8 ± 3.2
	Soxhlet	<LOQ	1627.5 ± 4.9	76.0 ± 4.6
Roasted pumpkin seed	Native	<LOQ	1149.4 ± 0.8 ^a,b^	20.1 ± 4.5 ^a,b^
	Macerate	<LOQ	1705.4 ± 21.9 ^b^	55.7 ± 8.6 ^b^
	Soxhlet	<LOQ	1458.9 ± 68.8	144.8 ± 7.9
Chia	Native	318.2 ± 12.1	609.5 ± 13.7 ^a,b^	<LOQ
	Macerate	313.2 ± 10.8	700.0 ± 4.5 ^b^	<LOQ
	Soxhlet	289.8 ± 6.3	843.7 ± 3.7	<LOQ
Linseed	Native	<LOQ	286.9 ± 6.5 ^a,b^	<LOQ
	Macerate	<LOQ	402.1 ± 6.7	<LOQ
	Soxhlet	<LOQ	436.5 ± 13.7	<LOQ
Hemp seed	Native	279.9 ± 6.3	679.3 ± 1.5 ^a,b^	<LOQ
	Macerate	286.9 ± 8.5	931.6 ± 36.0 ^b^	<LOQ
	Soxhlet	279.8 ± 6.3	332.8 ± 1.6	<LOQ

LOQ: limit of quantification; the values are represented as mean ± SD; means with different lowercase letters between various extracts indicate significant differences (*p* < 0.05).

**Table 4:** Hydroxycinnamic acid (HCA) content (µg/g).

Sample		Cinnamic Acid	p-Coumaric Acid	Caffeic Acid	Ferulic Acid	Hesperetic Acid	Synapic Acid	Dihydrocaffeic Acid	Phloretic ACID	Chlorogenic Acid
Raw peanut	Native	48.5 ± 4.5 ^a,b^	940.0 ± 57.7 ^a,b^	587.4 ± 24.3 ^a,b^	283.9 ± 7.0	551.9 ± 23.0	174.4 ± 10.9 ^a,b^	1087.4 ± 15.2 ^a,b^	499.3 ± 24.9 ^a,b^	224.3 ± 3.4 ^a^
	Macerate	25.9 ± 1.6 ^b^	288.3 ± 12.5	418.8 ± 18.1 ^b^	245.4 ± 12.7 ^c^	493.6 ± 16.9	109.2 ± 15.6 ^b^	743.4 ± 16.0 ^b^	383.4 ± 17.6 ^b^	153.8 ± 4.6 ^b^
	Soxhlet	34.9 ± 2.1	351.0 ± 16.3	643.1 ± 18.4	333.8 ± 13.0	531.7 ± 12.9	136.7 ± 8.1	1210.0 ± 52.7	852.1 ± 29.0	198.6 ± 19.6 ^a^
Roasted peanuts	Native	41.4 ± 1.9 ^a^	845.5 ± 132.6 ^a,b^	403.0 ± 24.0 ^a^	225.9 ± 45.4	557.0 ± 15.3	144.1 ± 20.1 ^a^	703.0 ± 31.7 ^a,b^	643.0 ± 35.1 ^a,b^	203.4 ± 21.3
	Macerate	49.8 ± 8.9	1093.4 ± 0.1	469.1 ± 16.1	242.7 ± 20.8	495.3 ± 22.1	146.8 ± 7.2 ^a^	1176.1 ± 0.9	439.5 ± 38.2 ^b^	202.9 ± 20.1
	Soxhlet	53.6 ± 4.7	1054.4 ± 8.0	516.0 ± 1.3	245.6 ± 11.3	589.0 ± 12.8	214.9 ± 32.3	1158.0 ± 7.5	920.1 ± 52.94	217.3 ± 18.0
Boiled peanuts	Native	48.0 ± 5.9 ^a,b^	463.8 ± 25.1 ^a^	414.8 ± 38.5	204.4 ± 12.9	584.1 ± 29.8	178.3 ± 21.2	828.6 ± 5.6 ^a,b^	696.2 ± 35.3	205.6 ± 15.5
	Macerate	34.5 ± 4.1 ^b^	615.6 ± 28.5 ^b^	401.7 ± 10.5 ^a^	193.5 ± 12.6	596.6 ± 30.4	145.6 ± 20.3 ^b^	1037.3 ± 77.1	619.3 ± 12.3	214.6 ± 7.7
	Soxhlet	114.6 ± 86.4	535.2 ± 9.5	486.5 ± 24.2	217.0 ± 24.8	569.4 ± 15.7	160.7 ± 23.5	1005.0 ± 23.0	694.3 ± 11.3	228.1 ± 22.1
Marinated peanuts	Native	42.3 ± 12.5 ^a^	551.1 ± 60.2	320.2 ± 40.0	168.6 ± 18.9	497.0 ± 23.8 ^a^	71.8 ± 71.3	936.2 ± 166.2 ^a^	512.3 ± 26.2 ^a^	161.5 ± 10.8
	Macerate	37.5 ± 2.1	550.4 ± 7.6	281.8 ± 29.7	144.8 ± 8.5	575.6 ± 26.9	75.5 ± 74.8	713.9 ± 20.4 ^b^	445.1 ± 37.3	148.7 ± 7.6
	Soxhlet	29.5 ± 0.6	550.7 ± 0.4	294.7 ± 42.2	147.7 ± 8.1	527.1 ± 62.7	64.8 ± 64.2	879.7 ± 102.1 ^a^	478.8 ± 63.6	155.8 ± 15.0
Raw almond	Native	66.4 ± 13.2 ^a^	135.9 ± 20.5 ^a,b^	<LOQ	117.6 ± 13.4 ^a,b^	<LOQ	<LOQ	331.7 ± 40.6 ^a,b^	200.4 ± 13.6 ^a^	122.9 ± 9.1
	Macerate	33.2 ± 2.4 ^b^	67.8 ± 15.2 ^b^	<LOQ	97.2 ± 9.9	<LOQ	<LOQ	531.1 ± 14.4	261.3 ± 11.5 ^a^	92.5 ± 9.4
	Soxhlet	54.8 ± 12.3 ^a^	99.4 ± 1.0	<LOQ	88.9 ± 9.0	<LOQ	<LOQ	518.5 ± 31.6	595.5 ± 48.4	118.5 ± 6.4
Roasted almond	Native	83.6 ± 31.2 ^a,b^	141.5 ± 27.1 ^a,b^	<LOQ	<LOQ	<LOQ	<LOQ	509.7 ± 60.1	252.0 ± 16.2 ^a,b^	155.2 ± 27.7 ^a^
	Macerate	55.0 ± 9.6 ^c^	106.6 ± 4.7	<LOQ	<LOQ	<LOQ	<LOQ	515.2 ± 35.3	724.4 ± 14.7 ^b^	139.7 ± 22.3 ^a^
	Soxhlet	37.7 ± 2.8	79.8 ± 16.4	<LOQ	<LOQ	<LOQ	<LOQ	495.8 ± 14.9	581.6 ± 22.2	97.7 ± 11.3
Boiled almond	Native	43.7 ± 0.3	91.9 ± 1.7 ^a^	<LOQ	<LOQ	<LOQ	<LOQ	293.9 ± 7.3	177.7 ± 17.1 ^a,b^	145.5 ± 6.6 ^a,b^
	Macerate	42.7 ± 3.9	96.1 ± 7.1 ^a^	<LOQ	<LOQ	<LOQ	<LOQ	254.2 ± 14.7	337.7 ± 27.0	52.8 ± 52.2 ^b^
	Soxhlet	38.3 ± 1.8	43.6 ± 43.6	<LOQ	<LOQ	<LOQ	<LOQ	300.8 ± 18.5	315.3 ± 14.0	104.0 ± 8.6
Raw hazelnuts	Native	46.6 ± 4.3	80.3 ± 15.9 ^a^	168.4 ± 5.6 ^a^	70.9 ± 11.5 ^a^	486.9 ± 23.8 ^a^	<LOQ	1545.9 ± 9.4 ^a,b^	283.8 ± 14.2 ^a,b^	84.2 ± 13.6 ^a^
	Macerate	58.3 ± 0.8	136.0 ± 18.7 ^b^	223.8 ± 18.2 ^b^	128.8 ± 27.4 ^b^	622.4 ± 13.5 ^b^	<LOQ	1815.0 ± 16.1	1003.9 ± 3.9 ^b^	137.2 ± 8.4 ^b^
	Soxhlet	52.5 ± 8.4	73.1 ± 9.4 ^a^	146.4 ± 13.2 ^a^	77.2 ± 4.2 ^a^	452.9 ± 12.2 ^a^	<LOQ	1863.5 ± 76.2	871.5 ± 22.4	97.9 ± 11.8 ^a^
Roasted hazelnuts	Native	90.3 ± 13.2 ^a,b^	150.8 ± 31.7 ^a,b^	233.6 ± 15.0 ^a,b^	<LOQ	657.9 ± 3.5 ^a,b^	<LOQ	165.3 ± 20.5 ^a^	447.1 ± 15.7 ^a^	148.1 ± 13.2 ^a^
	Macerate	49.4 ± 3.0	90.7 ± 9.7	178.0 ± 7.8	<LOQ	528.5 ± 17.4	<LOQ	209.5 ± 12.2	456.3 ± 30.2 ^a^	135.6 ± 6.9 ^a^
	Soxhlet	48.8 ± 3.2	81.3 ± 14.8	178.8 ± 8.4	<LOQ	466.6 ± 22.4	<LOQ	163.3 ± 15.2^a^	305.1 ± 0.8	96.2 ± 16.0
Walnut	Native	87.4 ± 6.5 ^a^	99.7 ± 0.1 ^a,b^	184.9 ± 0.1	100.9 ± 12.3	524.1 ± 4.5 ^a^	<LOQ	3055.9 ± 839.1 ^a^	1059.9 ± 103.5	205.6 ± 1.3
	Macerate	85.9 ± 3.5 ^a^	90.0 ± 2.6^b^	188.1 ± 0.1	99.1 ± 3.9	503.6 ± 8.6 ^a^	<LOQ	3294.3 ± 113.1 ^a^	981.6 ± 14.2	224.1 ± 1.1
	Soxhlet	100.3 ± 0.7	80.2 ± 2.2	170.3 ± 5.0	88.9 ± 3.7	442.7 ± 9.0	<LOQ	2477.6 ± 2.5	1011.3 ± 1.2	220.0 ± 1.1
Brazil nut	Native	58.3 ± 2.3 ^a,b^	171.9 ± 2.7 ^a,b^	276.1 ± 5.0 ^a,b^	181.9 ± 10.2 ^a,b^	857.4 ± 3.7 ^a,b^	235.6 ± 2.0 ^a,b^	1109.5 ± 3.8 ^a,b^	670.2 ± 12.7 ^a,b^	<LOQ
	Macerate	32.8 ± 2.9	91.7 ± 6.7	156.9 ± 13.6	120.6 ± 10.9	519.2 ± 2.8	135.4 ± 13.3	1267.1 ± 59.2	1286.4 ± 31.4 ^b^	<LOQ
	Soxhlet	33.5 ± 1.5	96.3 ± 4.6	176.5 ± 1.9	139.2 ± 12.1	520.4 ± 0.4	140.5 ± 3.4	1311.7 ± 0.0	1625.3 ± 4.1	<LOQ
Cashew raw	Native	29.6 ± 0.4	<LOQ	<LOQ ^a,b^	127.5 ± 0.0 ^a,b^	<LOQ	<LOQ	736.9 ± 10.3	369.5 ± 2.2 ^a,b^	200.3 ± 15.4
	Macerate	28.8 ± 0.3	64.6 ± 6.7 ^a^	115.0 ± 14.5 ^b^	96.2 ± 3.4 ^b^	<LOQ	<LOQ	149.9 ± 7.9 ^a^	883.1 ± 23.6	141.8 ± 10.2 ^a^
	Soxhlet	22.1 ± 13.0	<LOQ	207.4 ± 12.7	156.8 ± 11.3	<LOQ	<LOQ	848.8 ± 74.7	914.2 ± 19.5	222.9 ± 7.0
Roasted cashew	Native	35.0 ± 2.0	82.3 ± 0.6	139.6 ± 11.9	77.7 ± 4.4 ^a^	<LOQ	<LOQ	717.1 ± 9.0 ^a,b^	694.5 ± 6.6 ^a^	149.9 ± 1.7
	Macerate	30.2 ± 0.4	70.3 ± 3.7	140.0 ± 2.8	73.5 ± 3.5 ^a^	<LOQ	<LOQ	1038.8 ± 88.1	785.3 ± 11.4	161.0 ± 1.4
	Soxhlet	28.7 ± 3.0	87.6 ± 1.2	152.3 ± 6.7	106.7 ± 1.9	<LOQ	<LOQ	1067.4 ± 7.8	852.4 ± 19.7	162.2 ± 7.9
Pecan nut	Native	71.2 ± 3.5	109.2 ± 4.7 ^a^	<LOQ	100.0 ± 4.7 ^a^	<LOQ	<LOQ	2558.6 ± 143.7 ^a,b^	361.6 ± 12.8	153.4 ± 3.1 ^a,b^
	Macerate	75.7 ± 1.0	91.7 ± 8.6 ^b^	<LOQ	89.7 ± 2.3	<LOQ	<LOQ	3068.6 ± 112.2	373.3 ± 11.8	195.8 ± 7.6
	Soxhlet	68.7 ± 1.7	99.1 ± 3.7 ^a^	<LOQ	78.0 ± 4.0	<LOQ	<LOQ	2823.6 ± 58.7	369.1 ± 9.4	198.1 ± 5.7
Pine nuts	Native	43.8 ± 0.0 ^a^	<LOQ	<LOQ	<LOQ	<LOQ	<LOQ	229.5 ± 1.2	<LOQ	<LOQ
	Macerate	34.8 ± 2.6	<LOQ	<LOQ	<LOQ	<LOQ	<LOQ	217.9 ± 0.5	<LOQ	<LOQ
	Soxhlet	29.5 ± 1.3	<LOQ	<LOQ	<LOQ	<LOQ	<LOQ	199.8 ± 2.6	<LOQ	<LOQ
Roasted pistachios	Native	36.8 ± 14.4	<LOQ	<LOQ	98.5 ± 2.2	278.5 ± 17.9 ^a^	<LOQ	374.2 ± 29.7	387.5 ± 38.4	161.0 ± 21.0 ^a,b^
	Macerate	31.8 ± 2.1	<LOQ	<LOQ	87.8 ± 4.9	213.3 ± 6.1	<LOQ	397.4 ± 1.1	370.9 ± 10.4	138.3 ± 4.3
	Soxhlet	28.0 ± 1.4	<LOQ	<LOQ	89.2 ± 3.7	201.6 ± 8.9	<LOQ	442.6 ± 0.1	344.3 ± 1.5	127.1 ± 11.7
Sesame	Native	177.5 ± 6.2 ^a,b^	104.9 ± 11.5 ^a^	275.0 ± 14.8 ^a^	176.9 ± 4.9	538.0 ± 2.6	199.0 ± 2.7	1489.6 ± 19.6 ^a,b^	1643.6 ± 2.5 ^a,b^	1470.9 ± 6.7 ^a,b^
	Macerate	33.9 ± 2.5	129.6 ± 18.0 ^b^	247.9 ± 10.6 ^a^	219.4 ± 18.0	581.5 ± 3.8	175.8 ± 11.3	459.2 ± 4.3	754.8 ± 9.9	2059.4 ± 29.0
	Soxhlet	29.0 ± 5.1	97.7 ± 6.0 ^a^	165.6 ± 10.9	186.9 ± 6.9	518.8 ± 0.9	156.6 ± 2.7	455.7 ± 20.1	762.6 ± 11.3	2253.2 ± 25.7
Black sesame	Native	43.0 ± 4.8 ^a,b^	109.5 ± 6.8	202.2 ± 14.1	97.2 ± 1.5 ^a,b^	587.8 ± 10.0	166.3 ± 4.4	368.6 ± 13.3	<LOQ	266.4 ± 15.3 ^a,b^
	Macerate	125.5 ± 6.9 ^b^	104.2 ± 9.5	191.2 ± 7.4	158.0 ± 10.8 ^b^	604.0 ± 15.4	169.4 ± 8.8	440.9 ± 12.1	<LOQ	337.0 ± 24.5
	Soxhlet	153.8 ± 20.8	105.2 ± 8.5	199.1 ± 11.4	103.8 ± 7.9	589.7 ± 10.0	166.6 ± 6.2	375.8 ± 11.0	<LOQ	341.9 ± 24.3
Raw sunflower seed	Native	175.7 ± 18.7	157.6 ± 22.7	304.1 ± 25.3	1392.2 ± 68.2 ^a,b^	566.7 ± 7.5	163.5 ± 4.7	293.2 ± 6.2 ^a^	447.9 ± 27.9 ^a^	116,928.7 ± 192.7 ^a,b^
	Macerate	150.7 ± 13.0	155.2 ± 12.7	260.3 ± 18.8	1607.8 ± 65.1	544.4 ± 27.9	180.9 ± 16.1	251.9 ± 12.6	487.8 ± 24.5 ^a^	132,498.5 ± 329.8
	Soxhlet	154.8 ± 9.6	144.6 ± 8.6	269.3 ± 4.8	1599.2 ± 51.2	587.0 ± 5.2	166.8 ± 0.8	226.0 ± 22.7	615.7 ± 13.1	133,467.1 ± 257.3
Roasted sunflower seed	Native	120.3 ± 17.4 ^a^	116.8 ± 8.9	<LOQ	256.6 ± 13.9 ^a^	532.6 ± 3.5	173.7 ± 6.1	343.8 ± 23.5	370.6 ± 13.2 ^a^	87,715.2 ± 473.0 ^a,b^
	Macerate	63.3 ± 24.1 ^b^	115.4 ± 6.3	<LOQ	297.5 ± 16.0	559.8 ± 3.0	214.9 ± 29.3	314.5 ± 18.5	404.8 ± 26.8 ^a^	100,109.5 ± 670.4
	Soxhlet	114.0 ± 10.6 ^a^	118.5 ± 9.3	<LOQ	322.0 ± 28.6	607.2 ± 13.2	179.4 ± 5.4	305.8 ± 22.8	550.0 ± 16.8	105,336.3 ± 386.4
Raw pumpkin seed	Native	63.0 ± 2.0 ^a,b^	113.3 ± 7.2	<LOQ	<LOQ	<LOQ	<LOQ	335.7 ± 20.8 ^a,b^	362.6 ± 17.3 ^a,b^	182.5 ± 11.5 ^a,b^
	Macerate	38.3 ± 2.7	110.9 ± 6.7	<LOQ	<LOQ	<LOQ	<LOQ	496.3 ± 31.0	530.4 ± 20.3	117.8 ± 6.7
	Soxhlet	47.7 ± 1.5	118.1 ± 3.7	<LOQ	<LOQ	<LOQ	<LOQ	585.2 ± 11.0	685.9 ± 35.9	132.4 ± 6.6
Roasted pumpkin seed	Native	71.1 ± 1.1 ^a,b^	96.9 ± 7.3	<LOQ	<LOQ	<LOQ	<LOQ	263.4 ± 22.3 ^a^	307.0 ± 5.9 ^a,b^	250.1 ± 14.2 ^a,b^
	Macerate	41.5 ± 0.2	96.0 ± 5.4	<LOQ	<LOQ	<LOQ	<LOQ	447.4 ± 12.0 ^b^	463.9 ± 19.6	308.5 ± 5.6 ^b^
	Soxhlet	49.1 ± 14.9	103.8 ± 6.0	<LOQ	<LOQ	<LOQ	<LOQ	272.0 ± 14.5	466.1 ± 12.7	109.8 ± 9.7
Chia	Native	184.1 ± 4.7 ^a,b^	102.3 ± 7.4	214.4 ± 21.9	105.3 ± 16.1 ^a,b^	533.0 ± 26.1	<LOQ	<LOQ	539.9 ± 10.1 ^a,b^	417.1 ± 41.1 ^a^
	Macerate	103.6 ± 10.4 ^b^	103.7 ± 9.5	204.1 ± 11.5	89.9 ± 6.5	614.4 ± 29.5	<LOQ	<LOQ	1033.8 ± 43.0 ^b^	452.6 ± 25.2
	Soxhlet	72.9 ± 7.9	104.9 ± 10.5	189.4 ± 9.9	77.8 ± 8.2	532.5 ± 25.2	<LOQ	<LOQ	648.9 ± 12.0	564.7 ± 5.0
Linseed	Native	2998.0 ± 69.5 ^a,b^	108.8 ± 1.8	185.5 ± 8.7	114.8 ± 5.6	<LOQ	162.3 ± 7.0	240.5 ± 29.5	320.1 ± 20.1	170.5 ± 28.1
	Macerate	2585.8 ± 83.1	108.7 ± 2.8	200.3 ± 11.3	118.7 ± 6.0	<LOQ	177.0 ± 13.9	250.6 ± 16.8	357.5 ± 25.2	185.8 ± 25.0
	Soxhlet	2446.4 ± 16.5	112.0 ± 2.2	208.7 ± 19.8	125.1 ± 3.6	<LOQ	179.3 ± 7.9	256.4 ± 13.7	423.1 ± 9.2 ^a^	202.8 ± 11.0
Hemp seed	Native	<LOQ	105.9 ± 4.1	174.3 ± 6.8	83.9 ± 4.1 ^a^	<LOQ	<LOQ	402.8 ± 5.1 ^a^	398.4 ± 10.9	491.5 ± 17.7 ^a,b^
	Macerate	<LOQ	128.5 ± 13.2	178.7 ± 14.9	107.7 ± 12.7	<LOQ	<LOQ	549.4 ± 14.9 ^b^	325.3 ± 13.9	745.2 ± 33.6
	Soxhlet	<LOQ	103.2 ± 3.9	174.0 ± 6.0	92.7 ± 6.1	<LOQ	<LOQ	387.5 ± 20.3 ^a^	331.5 ± 4.2	742.7 ± 25.6

LOQ: limit of quantification; the values are represented as mean ± SD; means with different lowercase letters between various extracts indicate significant differences (*p* < 0.05).

**Table 5:** Content of flavonoid derivatives (µg/g).

Sample		Catechine	Epicatechine	Naringenine	Naringin	Rutin	Quercetin
Raw peanut	Native	6594.3 ± 137.9 ^a,b^	510.2 ± 16.3 ^a,b^	49.6 ± 18.4 ^a,b^	1163.0 ± 67.2 ^a,b^	2748.6 ± 66.9 ^a,b^	1118.8 ± 39.0
	Macerate	5329.5 ± 88.1 ^b^	640.5 ± 22.7 ^b^	<LOQ	710.3 ± 75.1	1748.2 ± 17.3 ^b^	1006.3 ± 22.3
	Soxhlet	7729.4 ± 96.2	821.2 ± 41.9	<LOQ	875.9 ± 85.5	2233.3 ± 110.1	1142.6 ± 43.2
Roasted peanuts	Native	5381.8 ± 13.5 ^a,b^	<LOQ ^a,b^	458.6 ± 30.1 ^a,b^	589.5 ± 44.7 ^a,b^	1919.1 ± 93.8 ^a^	1295.2 ± 26.2
	Macerate	7999.3 ± 158.1	42.2 ± 19.3 ^b^	384.9 ± 39.5 ^b^	868.4 ± 55.8	2200.0 ± 198.7	1142.3 ± 15.8 ^a^
	Soxhlet	7273.9 ± 101.4	22.6 ± 20.6	251.2 ± 38.9	745.9 ± 38.4	2063.3 ± 208.4	1356.2 ± 30.2
Boiled peanuts	Native	6379.1 ± 78.8 ^a^	510.3 ± 124.7 ^a,b^	<LOQ	670.6 ± 31.6	2199.9 ± 21.8	1289.2 ± 10.1
	Macerate	5881.4 ± 102.2 ^b^	729.4 ± 019.4 ^b^	<LOQ	592.2 ± 14.4	2143.4 ± 39.6	1386.8 ± 27.1
	Soxhlet	7115.2 ± 242.0 ^a^	948.0 ± 90.7	<LOQ	623.3 ± 6.9	2281.8 ± 57.0	1240.0 ± 33.2
Marinated peanuts	Native	5138.9 ± 84.7 ^a,b^	183.2 ± 49.0 ^a^	<LOQ	482.5 ± 8.6	2342.0 ± 134.5 ^a^	1253.9 ± 120.5
	Macerate	3102.0 ± 71.9 ^b^	217.7 ± 30.3 ^a^	<LOQ	401.7 ± 16.4	2546.0 ± 109.4 ^a^	1397.0 ± 26.4
	Soxhlet	4015.9 ± 238.4	322.6 ± 94.6	<LOQ	446.2 ± 1.5	3144.2 ± 98.1	1290.7 ± 61.8
Raw almond	Native	259.7 ± 10.8 ^a,b^	<LOQ	583.1 ± 17.9 ^a^	282.2 ± 30.4 ^a^	<LOQ	1439.2 ± 86.2 ^a^
	Macerate	354.6 ± 29.7	<LOQ	391.2 ± 4.8 ^b^	174.2 ± 9.1 ^b^	<LOQ	1018.4 ± 91.9 ^b^
	Soxhlet	410.3 ± 26.0	<LOQ	548.2 ± 35.6 ^a^	222.9 ± 13.4 ^a^	<LOQ	1291.0 ± 70.6 ^a^
Roasted almond	Native	486.0 ± 25.2	<LOQ	556.0 ± 24.6 ^a^	266.4 ± 26.2	<LOQ	1397.7 ± 87.5 ^a^
	Macerate	456.1 ± 15.5	<LOQ	495.4 ± 10.8	251.1 ± 26.0	<LOQ	1270.6 ± 92.7
	Soxhlet	408.1 ± 28.1	<LOQ	446.5 ± 19.4	194.9 ± 15.3	<LOQ	1208.9 ± 100.5
Boiled almond	Native	382.4 ± 12.9	<LOQ	<LOQ	94.6 ± 14.0 ^a,b^	<LOQ	1207.2 ± 67.1 ^a,b^
	Macerate	398.1 ± 28.6	<LOQ	<LOQ	75.2 ± 12.0 ^b^	<LOQ	1439.5 ± 102.7 ^b^
	Soxhlet	389.1 ± 28.9	<LOQ	<LOQ	166.5 ± 9.7	<LOQ	1309.3 ± 79.0
Raw hazelnuts	Native	378.6 ± 2.6 ^a^	277.5 ± 28.7 ^a^	413.0 ± 28.9 ^a^	285.6 ± 12.7 ^a^	1193.7 ± 81.3 ^a^	1136.1 ± 20.8 ^a,b^
	Macerate	503.9 ± 22.2 ^b^	340.6 ± 26.2	549.2 ± 28.5 ^b^	392.2 ± 14.1 ^b^	1376.3 ± 47.5 ^b^	1387.5 ± 18.3 ^b^
	Soxhlet	359.6 ± 10.6 ^a^	339.2 ± 6.6	381.0 ± 21.5 ^a^	280.8 ± 26.7 ^a^	1130.1 ± 69.0 ^a^	1592.2 ± 61.0
Roasted hazelnuts	Native	640.8 ± 14.2 ^a,b^	419.1 ± 7.5	582.7 ± 13.8 ^a,b^	359.5 ± 19.9	1459.6 ± 52.6 ^a,b^	1484.3 ± 58.4 ^a,b^
	Macerate	497.0 ± 13.6 ^b^	432.2 ± 12.2	445.3 ± 24.7	291.8 ± 17.5	1234.5 ± 78.3	1323.4 ± 42.0 ^b^
	Soxhlet	392.7 ± 8.8	434.0 ± 29.3	389.3 ± 26.8	295.4 ± 14.8	1168.8 ± 27.1	1683.3 ± 55.4
Walnut	Native	6530.4 ± 138.8 ^a^	1194.6 ± 106.6 ^a^	508.2 ± 20.6	1744.8 ± 17.9 ^a,b^	2593.5 ± 200.7 ^a,b^	1273.1 ± 44.7 ^a^
	Macerate	8052.1 ± 65.6 ^b^	1688.4 ± 78.9 ^b^	477.1 ± 6.3	1064.5 ± 22.6 ^b^	2082.5 ± 69.3 ^b^	1253.6 ± 47.8 ^a^
	Soxhlet	5927.4 ± 81.0 ^a^	1065.6 ± 48.0 ^a^	460.9 ± 26.4	1373.4 ± 45.3	1651.8 ± 78.4	1132.7 ± 22.4
Brazil nut	Native	684.7 ± 11.9 ^a^	475.6 ± 14.1 ^a^	670.9 ± 22.9 ^a,b^	344.7 ± 53.9 ^a,b^	<LOQ	1917.1 ± 98.9 ^a,b^
	Macerate	570.4 ± 15.4	559.4 ± 33.0 ^a^	455.6 ± 10.1	236.1 ± 14.7	<LOQ	1181.3 ± 13.9 ^b^
	Soxhlet	598.7 ± 19.4	671.2 ± 26.3	443.8 ± 22.2	230.3 ± 11.2	<LOQ	1231.2 ± 16.4
Raw cashew	Native	450.0 ± 4.7 ^a^	208.7 ± 12.4 ^a,b^	540.7 ± 34.6 ^a^	294.4 ± 18.9 ^a^	<LOQ	1306.2 ± 36.2 ^a,b^
	Macerate	477.2 ± 21.2 ^a^	290.1 ± 22.6	357.5 ± 25.6	171.9 ± 14.8 ^b^	<LOQ	1027.0 ± 50.3 ^b^
	Soxhlet	627.7 ± 79.7	326.5 ± 32.1	543.3 ± 64.7 ^a^	285.8 ± 1.4 ^a^	<LOQ	1444.4 ± 46.6
Roasted cashew	Native	359.0 ± 9.6 ^a,b^	273.2 ± 25.5	416.5 ± 39.1	<LOQ	<LOQ	1086.4 ± 16.5
	Macerate	579.4 ± 16.6	299.2 ± 13.0	395.9 ± 13.9	<LOQ	<LOQ	1051.7 ± 84.8
	Soxhlet	536.8 ± 9.6	330.3 ± 12.4	443.9 ± 20.2	<LOQ	<LOQ	1156.4 ± 35.2
Pecan nut	Native	2452.1 ± 122.3 ^a^	653.8 ± 12.8 ^a,b^	453.8 ± 25.3	406.3 ± 13.9	1229.4 ± 33.9 ^a^	1235.3 ± 28.5
	Macerate	3029.0 ± 85.2 ^b^	833.2 ± 10.8	447.5 ± 25.8	432.1 ± 14.1	1236.7 ± 39.8 ^a^	1339.9 ± 47.1
	Soxhlet	2633.2 ± 122.1 ^a^	827.4 ± 35.1	447.6 ± 18.9	418.5 ± 27.6	1053.1 ± 74.3	1224.0 ± 26.2
Pine nuts	Native	1111.5 ± 16.2 ^a,b^	299.5 ± 9.9 ^a^	<LOQ	224.5 ± 20.6	<LOQ	1297.5 ± 22.2 ^a,b^
	Macerate	1453.9 ± 50.7	418.3 ± 31.1 ^b^	<LOQ	248.6 ± 16.2	<LOQ	1531.0 ± 55.9 ^b^
	Soxhlet	1533.9 ± 56.2	261.5 ± 11.3 ^a^	<LOQ	214.8 ± 21.3	<LOQ	1411.0 ± 55.9
Roasted pistachios	Native	2041.4 ± 126.9 ^a^	269.9 ± 10.3 ^a,b^	432.1 ± 56.2	113.3 ± 18.8 ^a,b^	1145.6 ± 97.6 ^a^	1163.8 ± 134.9 ^a,b^
	Macerate	2073.3 ± 35.3 ^a^	435.2 ± 23.0 ^b^	455.4 ± 11.1	73.8 ± 12.7 ^b^	895.2 ± 39.6 ^b^	1276.7 ± 37.9
	Soxhlet	2397.7 ± 56.1	569.3 ± 18.7	486.0 ± 13.6	210.4 ± 16.0	1253.8 ± 32.2 ^a^	1303.0 ± 42.8
Sesame	Native	641.7 ± 23.6 ^a,b^	3847.8 ± 61.3 ^a,b^	5543.4 ± 62.8 ^a,b^	550.8 ± 47.5 ^a,b^	1952.5 ± 35.8 ^a,b^	1270.4 ± 60.9 ^a^
	Macerate	897.4 ± 64.6	2899.5 ± 16.1 ^b^	7456.9 ± 93.7	385.3 ± 22.3	2417.4 ± 95.8 ^b^	1395.4 ± 95.6 ^b^
	Soxhlet	903.0 ± 83.7	2097.6 ± 96.4	7632.9 ± 21.5	372.2 ± 18.7	1521.3 ± 93.3	1256.7 ± 85.6 ^a^
Black sesame	Native	513.5 ± 11.0 ^a^	135.8 ± 17.7 ^a^	615.0 ± 58.1 ^a,b^	359.5 ± 22.6 ^a^	1473.1 ± 13.4 ^a,b^	1407.8 ± 53.7
	Macerate	513.2 ± 26.6 ^a^	129.1 ± 15.5 ^a^	802.8 ± 15.8	423.1 ± 20.3	1662.5 ± 112.3 ^b^	1447.0 ± 25.2
	Soxhlet	414.6 ± 25.3	89.1 ± 13.9	735.2 ± 32.9	516.4 ± 15.1	1205.5 ± 56.4	1352.6 ± 6.1
Raw sunflower seed	Native	625.8 ± 20.0	<LOQ	553.6 ± 13.2	221.0 ± 15.7 ^a^	1298.2 ± 83.7 ^a^	1532.4 ± 75.7
	Macerate	671.9 ± 33.3	<LOQ	530.0 ± 5.5	462.2 ± 42.0 ^b^	1268.6 ± 66.8 ^a^	1584.4 ± 83.7
	Soxhlet	673.5 ± 48.4	<LOQ	537.2 ± 7.3	249.6 ± 14.1 ^a^	1443.8 ± 19.5	1588.7 ± 47.2
Roasted sunflower seed	Native	416.4 ± 34.5 ^a^	<LOQ	455.1 ± 16.7 ^a^	267.5 ± 13.7 ^a^	1446.8 ± 15.2 ^a^	1596.9 ± 73.3
	Macerate	441.4 ± 20.7 ^a^	<LOQ	445.0 ± 16.5 ^a^	436.6 ± 27.7 ^b^	1419.1 ± 23.6 ^a^	1459.1 ± 65.7
	Soxhlet	577.6 ± 41.8	<LOQ	573.9 ± 34.7	264.3 ± 37.9 ^a^	1314.7 ± 21.1	1489.5 ± 55.8
Raw pumpkin seed	Native	854.5 ± 56.1 ^a,b^	<LOQ ^a,b^	491.0 ± 20.2 ^a^	231.9 ± 20.5	<LOQ	903.5 ± 92.9 ^a,b^
	Macerate	1294.0 ± 116.1	142.7 ± 13.2	428.4 ± 13.8 ^a^	240.0 ± 20.3	<LOQ	1192.6 ± 41.1 ^b^
	Soxhlet	1239.3 ± 100.9	171.1 ± 15.3	308.6 ± 17.0	264.0 ± 11.8	<LOQ	1382.9 ± 59.3
Roasted pumpkin seed	Native	827.4 ± 61.0 ^a,b^	130.3 ± 14.7 ^a,b^	439.1 ± 13.3 ^a^	204.0 ± 10.7	<LOQ	1251.2 ± 27.8
	Macerate	988.9 ± 97.1	194.8 ± 14.8 ^b^	494.7 ± 13.2 ^a^	247.21 ± 18.8	<LOQ	1233.9 ± 56.1
	Soxhlet	1036.9 ± 37.6	233.3 ± 8.9	330.2 ± 12.7	224.8 ± 18.7	<LOQ	1162.8 ± 83.7
Chia	Native	3749.6 ± 355.6 ^a,b^	192.4 ± 8.4 ^a,b^	517.0 ± 31.7 ^a,b^	3726.7 ± 24.9 ^a^	1349.4 ± 49.1 ^a^	1219.8 ± 83.0 ^a^
	Macerate	4143.0 ± 109.4 ^b^	<LOQ	336.4 ± 15.7 ^b^	2317.0 ± 63.0 ^b^	1505.0 ± 39.9 ^b^	1413.5 ± 40.7
	Soxhlet	6061.5 ± 63.1	<LOQ	452.5 ± 11.0	3839.3 ± 30.5 ^a^	1317.6 ± 33.6 ^a^	1341.0 ± 63.0
Linseed	Native	568.2 ± 20.2 ^a,b^	608.2 ± 31.9 ^a^	16,093.0 ± 29.2 ^a,b^	203.9 ± 12.4	1277.9 ± 56.6	61,129.0 ± 391.2 ^a^
	Macerate	410.0 ± 13.9 ^b^	533.7 ± 36.7 ^b^	14,910.0 ± 577.1 ^b^	240.1 ± 11.0	1314.5 ± 38.4	54,474.4 ± 456.9 ^b^
	Soxhlet	721.0 ± 33.1	688.2 ± 33.9 ^a^	13,769.1 ± 378.6	244.5 ± 15.5	1282.4 ± 54.6	66,677.2 ± 481.9 ^a^
Hemp seed	Native	<LOQ	443.6 ± 25.0 ^a^	462.8 ± 14.6 ^a^	186.8 ± 15.2	<LOQ	<LOQ
	Macerate	<LOQ	465.0 ± 40.4 ^a^	292.5 ± 17.8 ^b^	206.3 ± 16.0	<LOQ	<LOQ
	Soxhlet	<LOQ	366.2 ± 7.7	454.9 ± 14.9 ^a^	190.2 ± 17.2	<LOQ	<LOQ

LOQ: limit of quantification; the values are represented as mean ± SD; means with different lowercase letters between various extracts indicate significant differences (*p* < 0.05).

## Data Availability

The original contributions presented in this study are included in the article and Supplementary Materials; further inquiries can be directed to the corresponding author.

## Supplementary Materials

The following supporting information can be downloaded at: https://www.mdpi.com/article/10.3390/foods14244191/s1. Table S1: Parameters of HPLC analysis; Figure S1: Pearson correlation analysis of HBAs in nuts (A) and seeds (B); Figure S2: Pearson correlation analysis of HCAs in nuts (A) and seeds (B).

## Abbreviations

The following abbreviations are used in this manuscript:

HPLC-DAD	High-performance liquid chromatography–diode array detector
PCA	Principal component analysis
HBAs	Hydroxybenzoic acids
HCAs	Hydroxycinnamic acids
TPC	Total phenolic content
FC	Folin–Ciocalteu
GA	Gallic acid
TFC	Total flavonoid content
C	Catechin
PTFE	Polytetrafluoroethylene
LOD	Limit of detection
LOQ	Limit of quantification
SPSS	Statistical Package for Social Science
ANOVA	One-way analysis of variance
LSD	Least significant difference
KMO	Kaiser–Meyer–Olkin
SOD	Superoxide dismutase
CAT	Catalase
GPx	Glutathione peroxidase
MDA	Malonyl dialdehyde
GAE	Gallic acid equivalents
EFSA	European Food Safety Authority
LDL	Low-density lipoprotein
DSS	Dextran sulfate sodium
CE	Catechin equivalents
QE	Quercetin equivalents
RUE	Rutin equivalents
NCDs	Non-communicable diseases
SPC	Sunflower press cake
DPPH	2,2-diphenyl-1-picrylhydrazyl
UN	United Nations

## References

[1] Wojdyło A., Turkiewicz I.P., Tkacz K., Nowicka P., Bobak Ł. (2022). Nuts as Functional Foods: Variation of Nutritional and Phytochemical Profiles and Their in Vitro Bioactive Properties. Food Chem. X.

[2] Dodevska M., Šobajić S., Đorđević B. (2015). Fibre and Polyphenols of Selected Fruits, Nuts and Green Leafy Vegetables Used in Serbian Diet. J. Serbian Chem. Soc..

[3] Dodevska M., Kukic Markovic J., Sofrenic I., Tesevic V., Jankovic M., Djordjevic B., Ivanovic N.D. (2022). Similarities and Differences in the Nutritional Composition of Nuts and Seeds in Serbia. Front. Nutr..

[4] Talwar B., Chopra R., Taneja N.K., Chand M., Homroy S., Dhiman A., Singh P.K., Chaudhary S. (2025). Use of Flaxseed Cake as a Source of Nutrients in the Food Industry and Possible Health Benefits- a Review. Food Prod. Process. Nutr..

[5] Ivanović N., Ilić T., Zrnić-Ćirić M., Todorović V., Đuričić I., Dabetić N. (2023). Agri-Food by-Products as a Source of Sustainable Ingredients for the Production of Functional Foods and Nutraceuticals. Arch. Pharm..

[6] Vichare S.A., Morya S. (2024). Exploring Waste Utilization Potential: Nutritional, Functional and Medicinal Properties of Oilseed Cakes. Front. Food Sci. Technol..

[7] Vicente-Zurdo D., Gómez-Mejía E., Morante-Zarcero S., Rosales-Conrado N., Sierra I. (2025). Analytical Strategies for Green Extraction, Characterization, and Bioactive Evaluation of Polyphenols, Tocopherols, Carotenoids, and Fatty Acids in Agri-Food Bio-Residues. Molecules.

[8] Sarkis J.R., Côrrea A.P.F., Michel I., Brandeli A., Tessaro I.C., Marczak L.D.F. (2014). Evaluation of the Phenolic Content and Antioxidant Activity of Different Seed and Nut Cakes from the Edible Oil Industry. J. Am. Oil Chem. Soc..

[9] Jasińska-Kuligowska I., Kuligowski M., Wyszyński M., Kidoń M. (2025). Upcycling of Sunflower and Sesame Press Cakes as Functional Ingredients in Cookies. Sustainability.

[10] Maciel L.G., Ribeiro F.L., Teixeira G.L., Molognoni L., Nascimento dos Santos J., Larroza Nunes I., Mara Block J. (2020). The Potential of the Pecan Nut Cake as an Ingredient for the Food Industry. Food Res. Int..

[11] Alvarez M.A., Alvarez M.A. (2014). Plant Secondary Metabolism. Chapter 3. Plant Biotechnology for Health: From Secondary Metabolites to Molecular Farming.

[12] Kumar N., Goel N. (2019). Phenolic Acids: Natural Versatile Molecules with Promising Therapeutic Applications. Biotechnol. Rep..

[13] Mihaylova D., Dimitrova-Dimova M., Popova A. (2024). Dietary Phenolic Compounds—Wellbeing and Perspective Applications. Int. J. Mol. Sci..

[14] Stevens-Barrón J.C., de la Rosa L.A., Wall-Medrano A., Álvarez-Parrilla E., Rodríguez-Ramirez R., Robles-Zepeda R.E., Astiazaran-García H. (2019). Chemical Composition and In Vitro Bioaccessibility of Antioxidant Phytochemicals from Selected Edible Nuts. Nutrients.

[15] Bouali I., Tsafouros A., Ntanos E., Albouchi A., Boukhchina S., Roussos P.A. (2023). Influence of Ripening Process on Pecan Nut (*Carya Illinoinensis*) Kernel Quality: Phenolic Profile, Antioxidant Activity, and Carbohydrate Composition. Horticulturae.

[16] Zardo I., de Espíndola Sobczyk A., Marczak L.D.F., Sarkis J. (2019). Optimization of Ultrasound Assisted Extraction of Phenolic Compounds from Sunflower Seed Cake Using Response Surface Methodology. Waste Biomass Valorization.

[17] Arrutia F., Binner E., Williams P., Waldron K.W. (2020). Oilseeds beyond Oil: Press Cakes and Meals Supplying Global Protein Requirements. Trends Food Sci. Technol..

[18] Ancuța P., Sonia A. (2020). Oil Press-Cakes and Meals Valorization through Circular Economy Approaches: A Review. Appl. Sci..

[19] Švarc-Gajić J., Rodrigues F., Moreira M.M., Delerue-Matos C., Morais S., Dorosh O., Silva A.M., Bassani A., Dzedik V., Spigno G. (2022). Chemical Composition and Bioactivity of Oilseed Cake Extracts Obtained by Subcritical and Modified Subcritical Water. Bioresour. Bioprocess..

[20] Garcia-Mendoza M.d.P., Espinosa-Pardo F.A., Savoire R., Etchegoyen C., Harscoat-Schiavo C., Subra-Paternault P. (2021). Recovery and Antioxidant Activity of Phenolic Compounds Extracted from Walnut Press-Cake Using Various Methods and Conditions. Ind. Crops Prod..

[21] Grahovac N., Aleksić M., Trajkovska B., Marjanović Jeromela A., Nakov G. (2025). Extraction and Valorization of Oilseed Cakes for Value-Added Food Components—A Review for a Sustainable Foodstuff Production in a Case Process Approach. Foods.

[22] Ojeda-Amador R.M., Salvador M.D., Fregapane G., Gómez-Alonso S. (2019). Comprehensive Study of the Phenolic Compound Profile and Antioxidant Activity of Eight Pistachio Cultivars and Their Residual Cakes and Virgin Oils. J. Agric. Food Chem..

[23] Ušjak L.J., Milutinović V.M., Đorđić Crnogorac M.J., Stanojković T.P., Niketić M.S., Kukić-Marković J.M., Petrović S.D. (2021). Barks of Three Wild *Pyrus* Taxa: Phenolic Constituents, Antioxidant Activity, and in Vitro and in Silico Investigations of α-Amylase and α-Glucosidase Inhibition. Chem. Biodivers..

[24] Ablat A., Mohamad J., Awang K., Shilpi J.A., Arya A. (2014). Evaluation of Antidiabetic and Antioxidant Properties of *Brucea javanica* Seed. Sci. World J..

[25] Pisinov B., Rakić R., Rakić S., Sekulić Z.Ž., Milićević T., Kulić G., Đurović S. (2024). Sustainable Utilization of *Novosadska variety* Buckwheat as Cultivated Biodiversity-Friendly Crop. Processes.

[26] Ros E., Singh A., O’Keefe J.H. (2021). Nuts: Natural Pleiotropic Nutraceuticals. Nutrients.

[27] George E.S., Daly R.M., Tey S.L., Brown R., Wong T.H.T., Tan S.-Y. (2022). Perspective: Is It Time to Expand Research on “Nuts” to Include “Seeds”? Justifications and Key Considerations. Adv. Nutr..

[28] Ros E. (2015). Nuts and CVD. Br. J. Nutr..

[29] Liu H.-Y., Liu Y., Li M.-Y., Ge Y.-Y., Geng F., He X.-Q., Xia Y., Guo B.-L., Gan R.-Y. (2022). Antioxidant Capacity, Phytochemical Profiles, and Phenolic Metabolomics of Selected Edible Seeds and Their Sprouts. Front. Nutr..

[30] Čakar U., Čolović M., Milenković D., Pagnacco M., Maksimović J., Krstić D., Đorđević B. (2025). Strawberry and Drupe Fruit Wines Antioxidant Activity and Protective Effect Against Induced Oxidative Stress in Rat Synaptosomes. Antioxidants.

[31] Lafay S., Gil-Izquierdo A. (2008). Bioavailability of Phenolic Acids. Phytochem. Rev..

[32] Kornsteiner M., Wagner K.-H., Elmadfa I. (2006). Tocopherols and Total Phenolics in 10 Different Nut Types. Food Chem..

[33] Pająk P., Socha R., Gałkowska D., Rożnowski J., Fortuna T. (2014). Phenolic Profile and Antioxidant Activity in Selected Seeds and Sprouts. Food Chem..

[34] Mingrou L., Guo S., Ho C.-T., Bai N. (2022). Review on Chemical Compositions and Biological Activities of Peanut (*Arachis hypogeae* L.). J. Food Biochem..

[35] Woźniak M., Waśkiewicz A., Ratajczak I. (2022). The Content of Phenolic Compounds and Mineral Elements in Edible Nuts. Molecules.

[36] Zhang Y., Xiao H., Lv X., Wang D., Chen H., Wei F. (2022). Comprehensive Review of Composition Distribution and Advances in Profiling of Phenolic Compounds in Oilseeds. Front. Nutr..

[37] Kumar P., Székely D., Szalóki-Dorkó L., Kereszturi J., Máté M. (2023). Phenolic profile, color parameters and antioxidant activity of walnut kernel extracts as influenced by different time and temperature during extraction. Prog. Agric. Eng. Sci..

[38] Popa R.-G., Bălăcescu A., Popescu L.G. (2023). Organic Walnut Cultivation in Intensive and Super-Intensive System—Sustainable Investment. Case Study: Gorj County, Romania. Sustainability.

[39] EFSA Panel on Dietetic Products N. (2011). and A. (NDA). Scientific Opinion on the Substantiation of Health Claims Related to Walnuts and Maintenance of Normal Blood LDL-Cholesterol Concentrations (ID 1156, 1158) and Improvement of Endothelium-Dependent Vasodilation (ID 1155, 1157) Pursuant to Article 13(1) of Regulation (EC) No 1924/2006. EFSA J..

[40] Heleno S.A., Martins A., Queiroz M.J., Ferreira I.C. (2015). Bioactivity of phenolic acids: Metabolites versus parent compounds: A review. Food Chem..

[41] Alqudah S., Claesen J. (2024). Mechanisms of gut bacterial metabolism of dietary polyphenols into bioactive compounds. Gut Microbes.

[42] Amić A., Lučić B., Marković Z., Amić D. (2016). Carboxyl Group as a Radical Scavenging Moiety: Thermodynamics of 2H+/2e– Processes of Phloretic Acid. Croat. Chem. Acta.

[43] Özcan M.M., Yılmaz F.G., Uslu N., Kulluk D.A., Dursun N., Yılmaz H. (2024). Determination of Bioactive Compounds, Phenolic Contents, Fatty Acid and Biogenic Element Profiles of the Seeds of Sunflower (*Helianthus annuus* L.) Genotypes. Food Humanit..

[44] Žilić S., Maksimović Dragišić J., Maksimović V., Maksimović M., Basić Z., Crevar M., Stanković G. (2010). The content of antioxidants in sunflower seed and kernel. Helia.

[45] Náthia-Neves G., Alonso E. (2021). Valorization of Sunflower By-Product Using Microwave-Assisted Extraction to Obtain a Rich Protein Flour: Recovery of Chlorogenic Acid, Phenolic Content and Antioxidant Capacity. Food Bioprod. Process..

[46] Akram N.A., Shafiq F., Ashraf M. (2018). Peanut (*Arachis hypogaea* L.): A Prospective Legume Crop to Offer Multiple Health Benefits Under Changing Climate. Compr. Rev. Food Sci. Food Saf..

[47] Salamatullah A.M., Alkaltham M.S., Özcan M.M., Uslu N., Hayat K. (2021). Effect of Maturing Stages on Bioactive Properties, Fatty Acid Compositions, and Phenolic Compounds of Peanut (*Arachis hypogaea* L.) Kernels Harvested at Different Harvest Times. J. Oleo Sci..

[48] Zhang Y., Zhu W., Jin Y., Xu J., Zhou W., Shen T., Yang A., Wu Z., Chen H. (2024). Effect of Boiling and Roasting Treatments on the Nutrients, Lipid Quality, and Flavor of Peanuts. Food Sci. Nutr..

[49] Zhang Y., Zhu J., Wu D., Liu X. (2024). Effects of heat treatment on nutritional profiles and antioxidant activity of peanuts. J. Food Saf. Food Qual..

[50] de Camargo A.C., Regitano-d’Arce M.A.B., Rasera G.B., Canniatti-Brazaca S.G., do Prado-Silva L., Alvarenga V.O., Sant’Ana A.S., Shahidi F. (2017). Phenolic Acids and Flavonoids of Peanut By-Products: Antioxidant Capacity and Antimicrobial Effects. Food Chem..

[51] Zayed A., Abdelkareem S., Talaat N., Abdel Dayem D., Farag M.A. (2025). Tannin in foods: Classification, Dietary Sources, and Processing Strategies to Minimize Anti-Nutrient Effects. Food Bioprocess Technol..

[52] Taş N.G., Gökmen V. (2017). Phenolic compounds in natural and roasted nuts and their skins: A brief review. Curr. Opin. Food Sci..

[53] Kulczyński B., Kobus-Cisowska J., Taczanowski M., Kmiecik D., Gramza-Michałowska A. (2019). The Chemical Composition and Nutritional Value of Chia Seeds-Current State of Knowledge. Nutrients.

[54] Biswas S., Islam F., Imran A., Zahoor T., Noreen R., Fatima M., Zahra S.M., Asif Shah M. (2023). Phytochemical Profile, Nutritional Composition, and Therapeutic Potentials of Chia Seeds: A Concise Review. Cogent Food Agric..

[55] Knez Hrnčič M., Ivanovski M., Cör D., Knez Ž. (2020). Chia Seeds (*Salvia hispanica* L.): An Overview—Phytochemical Profile, Isolation Methods, and Application. Molecules.

[56] Przybylska-Balcerek A., Frankowski J., Graczyk M., Niedziela G., Sieracka D., Wacławek S., Sázavská T.H., Buśko M., Szwajkowska-Michałek L., Stuper-Szablewska K. (2024). Profile of Polyphenols, Fatty Acids, and Terpenes in Henola Hemp Seeds Depending on the Method of Fertilization. Molecules.

[57] Naseem N., Ahmad M.F., Imam N., Ahsan H., Siddiqui W.A. (2023). The Potential of Esculin as a Therapeutic Modality in Diabetes Mellitus and Its Complications. Hum. Nutr. Metab..

[58] Ju S., Tan Y., Wang Q., Zhou L., Wang K., Wen C., Wang M. (2024). Antioxidant and anti inflammatory effects of esculin and esculetin (Review). Exp. Ther. Med..

[59] Ali N.A., Elsayed G.H., Mohamed S.H., Abd Elkarim A.S., Aly M.S., Elgamal A.M., Elsayed W.M., El-Newary S.A. (2024). Chia Seed (*Salvia hispanica*) Attenuates Chemically Induced Lung Carcinomas in Rats through Suppression of Proliferation and Angiogenesis. Pharmaceuticals.

[60] Zhang K., Ma J., Gangurde S.S., Hou L., Xia H., Li N., Pan J., Tian R., Huang H., Wang X. (2022). Targeted metabolome analysis reveals accumulation of metabolites in testa of four peanut germplasms. Front. Plant Sci..

[61] Gavril R.N., Stoica F., Lipșa F.D., Constantin O.E., Stănciuc N., Aprodu I., Râpeanu G. (2024). Pumpkin and Pumpkin By-Products: A Comprehensive Overview of Phytochemicals, Extraction, Health Benefits, and Food Applications. Foods.

[62] Voučko B., Novotni D., Balbino S., Mustač N.Č., Drakula S., Dujmić F., Habuš M., Jarni K., Ćurić D. (2022). Utilization of pumpkin seed oil cake and proso millet flour in enhancing gluten-free bread quality. J. Food Process. Preserv..

[63] Vasileiou C., Dimoula M., Drosou C., Kavetsou E., Stergiopoulos C., Gogou E., Boukouvalas C., Krokida M. (2025). Valorization of Edible Oil Industry By-Products Through Optimizing the Protein Recovery from Sunflower Press Cake via Different Novel Extraction Methods. AgriEngineering.

[64] Mondor M. (2023). Chia (*Salvia hispanica*) Seed Oil Extraction By-Product and Its Edible Applications. Food Rev. Int..

[65] Suleman D., Bashir S., Hassan Shah F.U., Ikram A., Zia Shahid M., Tufail T., Ahmad Khan A., Ahsan F., Ambreen S., Raya A. (2023). Nutritional and functional properties of cookies enriched with defatted peanut cake flour. Cogent Food Agric..

