# Supplementary material for: Phenolic Composition in Native and Defatted Nuts and Seeds from the Serbian Market: Analytical Insights and Functional Potential
Source: Foods. 2025 Dec 6;14(24):4191. doi: 10.3390/foods14244191 (PMC12731319; doi:10.3390/foods14244191)
Supplement: Supplementary file 1 [file foods-14-04191-s001.zip › foods-3976944-supplementary.pdf]

Supplementary material

Phenolic Composition in Native and Defatted Nuts and Seeds from the Serbian Market: Analytical Insights and Functional Potential

Margarita Dodevska[1], Nevena Ivanović[2], Sanja Đurović[3], Boris Pisinov[3], Uroš Čakar[2], Jelena Kukić Marković[4,*]

Corresponding author:
Jelena Kukić-Marković
University of Belgrade - Faculty of Pharmacy,
Department of Pharmacognosy.
Vojvode Stepe 450. Belgrade. Serbia
jelena.kukic@pharmacy.bg.ac.rs

Table S1. Parameters of HPLC analysis

	Retention time (min)	Standards	Equation	r^2	LOD (μg/mL)	LOQ (μg/mL)
1.	8.43	Gallic acid	y=26764.3*x-38937.3	0.9980	0.04	0.11
2.	13.99	Protocatechuic acid	y=15681.0*x-21814.4	0.9970	0.04	0.13
3.	19.92	Esculin	y=22044.6*x-4327.5	0.9968	0.02	0.07
4.	20.48	Catechin	y=8838.2*x-18084.7	0.9956	0.04	0.12
5.	21.60	Dihydrocaffeic acid	y=9114.8*x-8525.4	0.9974	0.07	0.21
6.	23.87	Chlorogenic acid	y=34176.2*x-19130.7	0.9979	0.03	0.09
7.	25.41	Caffeic acid	y=61866.3*x-57537.3	0.9969	0.01	0.03
8.	27.02	Epicatechin	y=6239.4*x-2977.6	0.9947	0.05	0.14
9.	27.53	Syringic acid	y=33501.9*x+8054.3	0.9967	0.02	0.07
10.	28.57	Phloretic acid	y=6416.0*x-9993.6	0.9964	0.04	0.11
11.	32.33	*p*-Coumaric acid	y=54077.2*x-27210.4	0.9965	0.02	0.07
12.	34.61	Ferulic acid	y=53968.4*x-24301.4	0.9967	0.02	0.06
13.	35.57	Sinapic acid	y=52797.1*x-41653.3	0.9974	0.01	0.03
14.	36.82	Hesperetic acid	y=61379.2*x-179752	0.9938	0.02	0.06
15.	40.10	Naringin	y=19742.7*x-20902.7	0.9970	0.05	0.14
16.	41.10	Rutin	y=11556.0*x-75733.2	0.9937	0.04	0.12
17.	46.45	*trans*-Cinnamic acid	y=84562.4*x-11197.8	0.9965	0.01	0.02
18.	47.25	Quercetin	y=10970.7*x-72104.0	0.9956	0.05	0.17
19.	47.54	Naringenin	y=49686.2*x-122998	0.9965	0.03	0.08

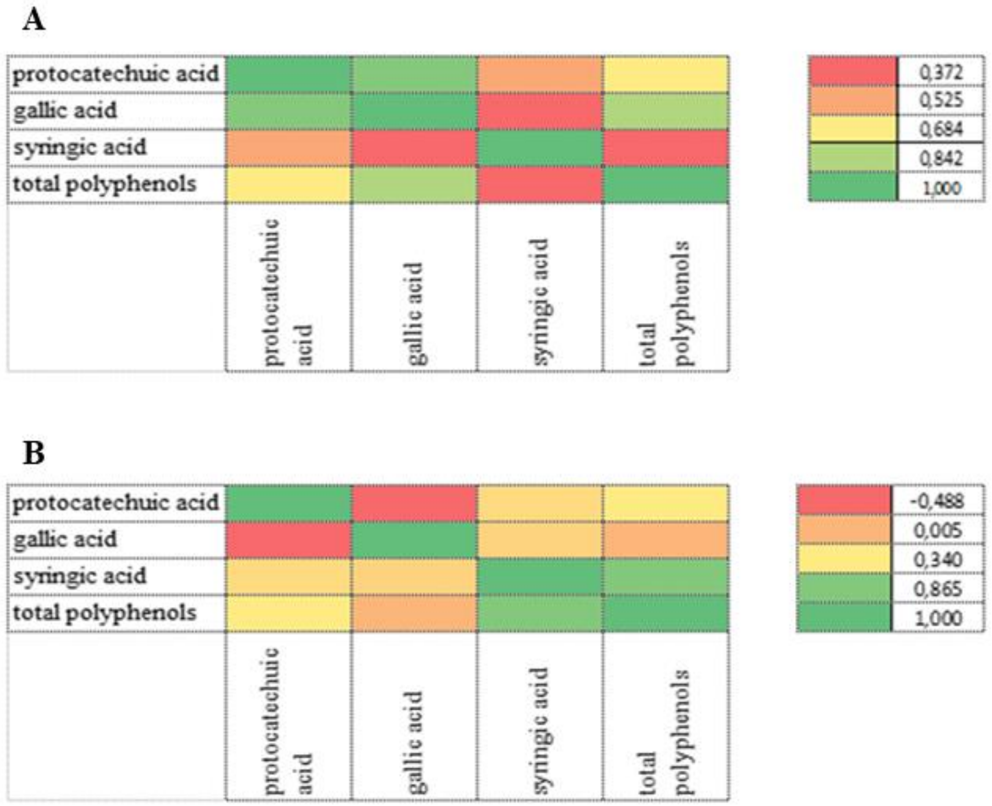

Figure S1. Pearson correlation HBAs in nuts (A) and seeds (B).

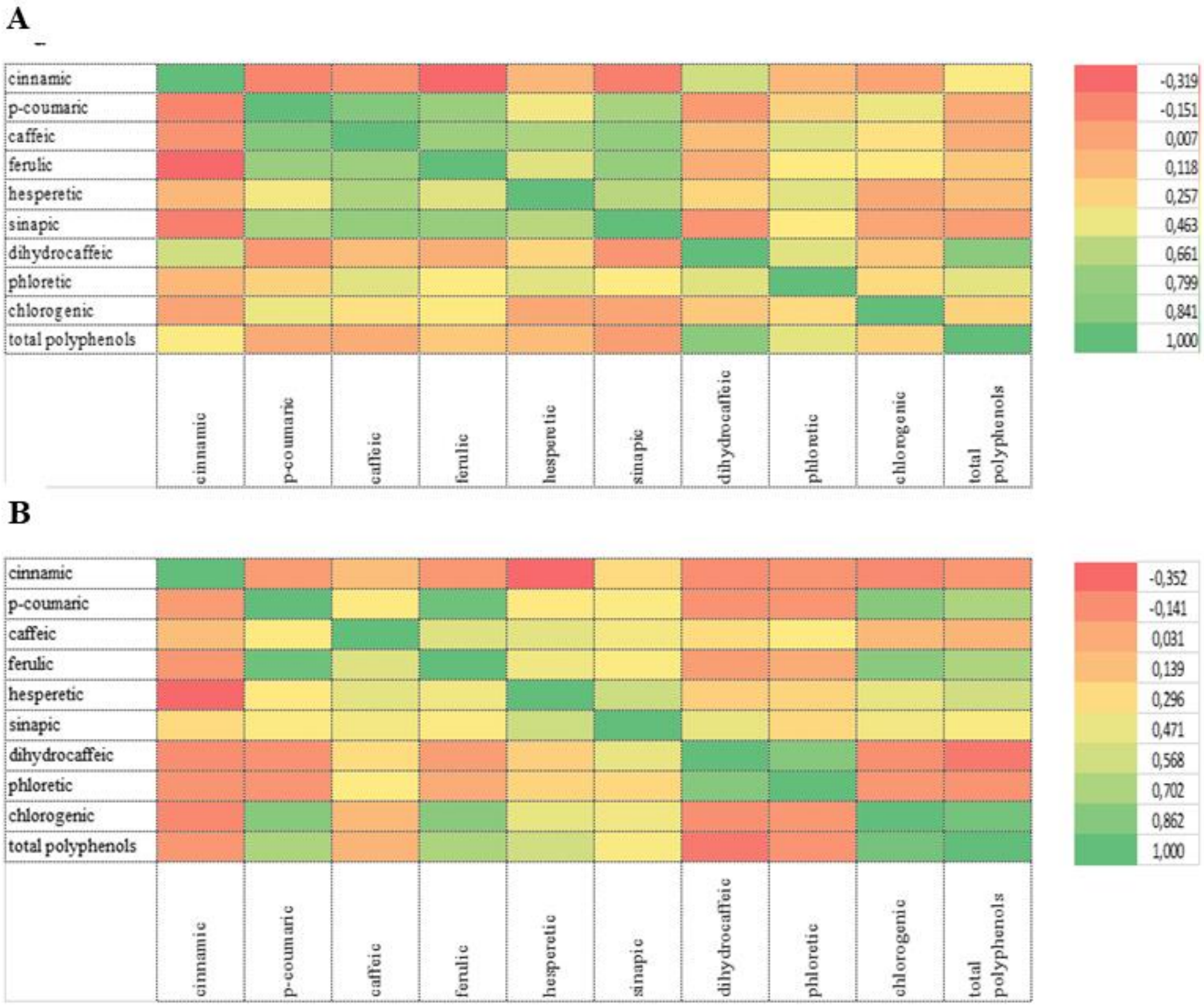

Figure S2. Pearson correlation HCAs in nuts (A) and seeds (B).
